# Streamflow prediction using an integrated methodology based on convolutional neural network and long short-term memory networks

**DOI:** 10.1038/s41598-021-96751-4

**Published:** 2021-09-01

**Authors:** Sujan Ghimire, Zaher Mundher Yaseen, Aitazaz A. Farooque, Ravinesh C. Deo, Ji Zhang, Xiaohui Tao

**Affiliations:** 1 School of Sciences, University of Southern Queensland, Toowoomba, QLD 4350 Australia; 2 New era and development in civil engineering research group, Scientific Research Center, Al-Ayen University, Thi-Qar, 64001 Iraq; 3 College of Creative Design, Asia University, Taichung City, Taiwan; 4 Faculty of Sustainable Design Engineering, University of Prince Edward Island, Charlottetown, PE C1A4P3 Canada

**Keywords:** Hydrology, Civil engineering, Environmental impact, Hydrology

## Abstract

Streamflow (*Q*_*flow*_) prediction is one of the essential steps for the reliable and robust water resources planning and management. It is highly vital for hydropower operation, agricultural planning, and flood control. In this study, the convolution neural network (CNN) and Long-Short-term Memory network (LSTM) are combined to make a new integrated model called CNN-LSTM to predict the hourly *Q*_*flow*_ (short-term) at Brisbane River and Teewah Creek, Australia. The CNN layers were used to extract the features of *Q*_*flow*_ time-series, while the LSTM networks use these features from CNN for *Q*_*flow*_ time series prediction. The proposed CNN-LSTM model is benchmarked against the standalone model CNN, LSTM, and Deep Neural Network models and several conventional artificial intelligence (AI) models. *Q*_*flow*_ prediction is conducted for different time intervals with the length of 1-Week, 2-Weeks, 4-Weeks, and 9-Months, respectively. With the help of different performance metrics and graphical analysis visualization, the experimental results reveal that with small residual error between the actual and predicted *Q*_*flow*_, the CNN-LSTM model outperforms all the benchmarked conventional AI models as well as ensemble models for all the time intervals. With 84% of *Q*_*flow*_ prediction error below the range of 0.05 m^3^ s^−1^, CNN-LSTM demonstrates a better performance compared to 80% and 66% for LSTM and DNN, respectively. In summary, the results reveal that the proposed CNN-LSTM model based on the novel framework yields more accurate predictions. Thus, CNN-LSTM has significant practical value in *Q*_*flow*_ prediction.

## Introduction

Accurate streamflow (*Q*_*flow*_) prediction is crucial for efficient water management tasks, such as improving the efficiency of hydroelectricity generation, irrigation planning and flood management^1^. However, because of the nonlinear behaviour of the streamflow time series, streamflow prediction remains one of the very difficult matters in the field of hydrological sciences^2,3^. In addition, the accurate prediction of *Q*_*flow*_ can contribute to several advantages for water resources project operation, efficient programming for flood monitoring, scheduling for reservoir operation, and several other hydrological processes. Therefore, the prediction of *Q*_*flow*_ is essential in the field of hydrological engineering^4^.

Several models have been used in the past research for the development of *Q*_*flow*_ prediction model in order to increase the accuracy in prediction. Stochastic models like, Auto Regressive (AR)^5^, Auto Regressive Moving Average (ARMA)^6^ and Autoregressive Moving Average with Exogenous Inputs (ARMAX)^7^, have been used for *Q*_*flow*_ prediction based on the time series^8^. These statistical models analyze the time series dataset for the goal of developing a reliable technology for simulating the streamflow using classical statistics. However, those models have shown limitations to capture the nonlinear characteristics of the *Q*_*flow*_. On the other hand, Artificial Intelligence (AI) based data-driven models such as Artificial Neural Network (ANN)^9,10^, Support Vector Machine (SVM)^11–13^, Extreme Learning Machine (ELM)^14,15^, Fuzzy Neural Network (FNN)^16,17^ and Genetic Programming (GP)^18–20^, have proven superior in modelling processes compared to the stochastic model. These AI models have demonstrated an excellent capacity in the field of hydrology owing to their potential in solving the mimicking the associated non-linearity and non-stationarity in the hydrological processes and reported a successful implementation for *Q*_*flow*_ process simulation^21–23^. For time series forecasting, it is important to abstract the correlated lagged *Q*_*flow*_ for building any data driven predictive model^24^.

Among several massively employed AI models in the field of hydrology, ANN model is the one for streamflow prediction^25^, which imitates the operation of biological neuron and can solve the associated nonlinearity phenomenal time-series data. One of the earliest conducted studies, Zealand et al.^26^ utilized ANN model to simulate *Q*_*flow*_ to a portion of the Winnipeg River system in Northwest Ontario, Canada. Authors concluded that the employed ANN model is superior to the conventional Winnipeg Flow Forecasting model (WIFFS) in term of the prediction capacity. Kerh and Lee^27^ predicted the *Q*_*flow*_ at the downstream of catchment using the data of upstream historical data. The research was conducted on the basis of flood forecasting due to the data non-availability at the downstream. The research evidenced the potential of the ANN over the classical Muskingum model. In another study, Adamowski and Sun^28^ developed ANN model coupled with discrete wavelet transform (DWT-ANN) for *Q*_*flow*_ prediction and found that DWT-ANN model outperformed the standalone ANN model. Demirel et al.^29^ studied the issue of flow prediction based on the soil and water assessment tool (SWAT) and ANN models; ANN shows better performance in peak flow prediction compared to SWAT.

Over the literature, several AI models introduced for the streamflow modelling such as support vector regression (SVR), adaptive neuro fuzzy inference system (ANFIS), extreme learning machine (ELM), random forest (RF), and their hybridized version with several optimization algorithms^30^. SVR model was used for long term (monthly) as well as short-term (hourly) *Q*_*flow*_ prediction and shown a better performance than ANFIS and GP^31,32^. Atiquzzaman and Kandasamy^33^ employed ELM model for streamflow prediction for two different catchment sizes from two different climatic conditions and benchmarked it with SVR and GP. The results showed that the prediction accuracy was increased, and computational time was minimised. ELM has been further employed by^34^ to predict mean *Q*_*flow*_ water level for three hydrological sites in eastern Queensland (Gowrie Creek, Albert, and Mary River).

**Figure 1:**
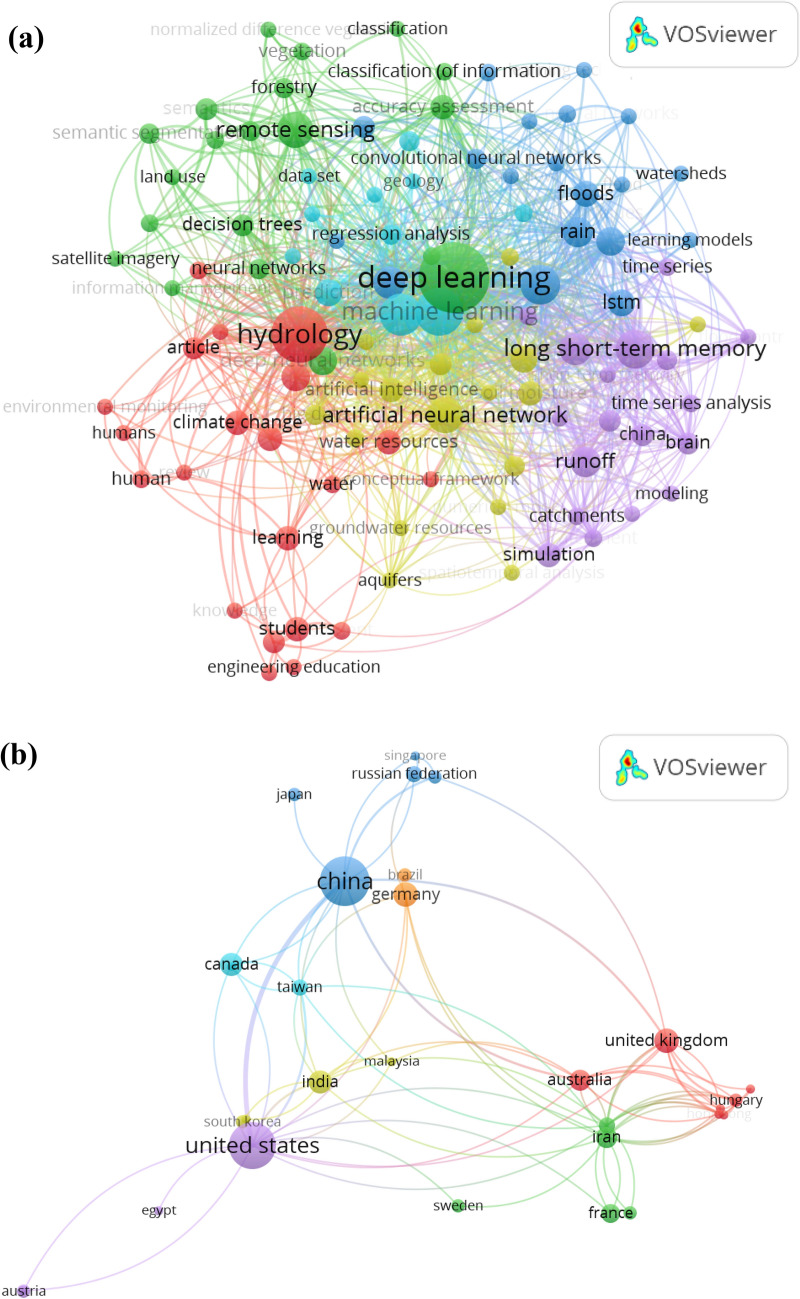
(**a**) The reported keywords occurrence (107 keywords) over the literature on the implementation of the deep learning models within the research domain of hydrology, (**b**) The investigated region around the globe.

Nevertheless, the implementation of AI models in the prediction of *Q*_*flow*_ are not consistent and it is difficult to conclude which method is superior. Additionally, the AI model, like the ANN model, has some limitations such as learning divergence, shortcoming in the generalizing performance, trapping in the local minimum and over-fitting problems^35^. Whereas, SVR model seems to be overcoming some drawbacks of ANN, however, requires a long simulation time because of the kernel function (penalty factor and kernel width)^13^. Hence, if the data complexity is high, the AI models (e.g., ANN, SVR, ELM, ANFIS, etc.) may fail to learn all the conditions effectively. The motivation on the new discovery for new and robust machine learning models is still ongoing in the field of hydrology. In the recent research, new AI models represented by deep learning (DL) models have been developed for *Q*_*flow*_ simulation. Various DL architectures (Deep Neural Network [DNN], Convolutional Neural Network [CNN] and Long Short-Term Memory [LSTM]) have been developed and widely used in the time-series prediction of solar radiation, wind, stock price etcetera^36,37^. These DL models such as the potential in handling highly stochastic datasets and abstracting the internal physical mechanism^38^. In more representative manner, Fig. 1 was generated using the VOSviewer software to exhibit the major keywords occurrence within Scopus database on the implementation of DL models in the field of hydrology in addition to the countries where the researches were adopted.

This study offers a deep learning model based on the integration of CNN and LSTM, where the CNN model is applied to extract the intrinsic features of the *Q*_*flow*_ time series while LSTM model utilizes the feature extracted by CNN for *Q*_*flow*_ prediction. The reason to use the CNN-LSTM for the prediction of *Q*_*flow*_ is to utilize the nonlinear processing capacity of CNN to obtain precise short-term *Q*_*flow*_ prediction accuracy. Moreover, in CNN-LSTM model, CNN is used to remove noise and to take into account the correlation between lagged variables of *Q*_*flow*_*,* LSTM models temporal information and maps time series into separable spaces to generate predictions. This CNN-LSTM model has been used previously in various areas; in the field of natural language processing, emotions were analyzed using the CNN-LSTM model with text input^39^; in the field of speech processing, voice search tasks were done using the CLDNN model combining CNN, LSTM, and DNN^40^; in the field of video processing, a model combining CNN and Bi-directional LSTM was designed to recognize human action in video sequences^41^; in the field of medical field, CNN-LSTM was developed to accurately detect arrhythmias in the electrocardiogram (ECG)^42^; in the field of industrial area, convolutional bi-directional long short-term memory network was designed to predict tool wear^43^. Furthermore, in time series application CNN-LSTM model was developed for efficient prediction of residential energy consumption^44,45^, solar radiation forecasting^46^, wind speed prediction^47^ and stock price prediction^48^. In this study the prediction of *Q*_*flow*_ is done on hourly basis for two hydrological catchments (Brisbane River:26.39° S 152 22° E and Teewah Creek: 26.16° S 153.03° E) in Australia. The main aim of the current research is to inspect the prediction capacity of several DL models in modelling hourly *Q*_*flow*_ and compare the DL model performance (CNN-LSTM, LSTM, CNN, DNN) with other AI models (Multilayer Perceptron [MLP], ELM) as well as ensemble models (Decision Tree [DT], Gradient Boosting Regression [GBM], Extreme Gradient Boosting [XGB] and Multivariate Adaptive Regression Splines [MARS]). This investigation is considered one the earliest in the Australian region that is conducted on the examination of the deep learning, conventional AI models and ensemble models for the problem of streamflow prediction.

## Theoretical overview

The theoretical overview of the deep learning model, CNN, LSTM, DNN and CNN-LSTM is presented in this section. The theoretical explanation of the MLP^49^, GBM^50^, ELM^51^, XGB^52^, DT^53^, MARS^54^ and RFR^55^ are all elucidated elsewhere since they are well-known conventional AI models (MLP, ELM) and ensemble methodologies (GBM, XGB, DT, MARS and RFR).

### Convolutional neural network

**Figure 2:**
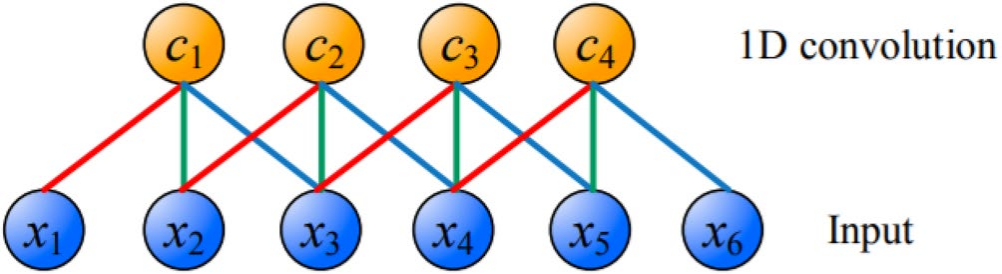
The 1-dimensional convolution operation. Symbol as per “Theoretical overview” section.

CNN model^56,57^ differs from MLP by relying on the weight sharing concept. In literatures, three types of CNN networks are found, one-dimensional CNN (Conv1D), two-dimensional CNN (Conv2D) and three-dimensional CNN (Conv3D). In Conv1D, the convolution kernels move in one direction. The input and output data of Conv1D is 2-dimensional^58^. Mainly used for time series data^59^, the Conv1D has powerful capability of feature extraction: the non-linear features hidden in the raw data can be automatically extracted by the alternating convolutional layer and pooling layer in the Conv1D, and the adaptive feature learning is completed at the fully-connected layer. In this way, the Conv1D algorithm eliminates the manual process of feature extraction in traditional algorithms and end-to-end information processing is realized^60^. Conv2D is the first standard CNN introduced in the Lenet-5 architecture^61^. Conv2D is usually used for image data^62^. It is called Conv2D because the convolution kernels slide along the data in 2 dimensions. The input and output data of Conv2D is 3-dimensional, for instance, in image classifications CNN can detect edges, color distribution, etc. in an image, making these networks very powerful in image classification and other similar data containing spatial characteristics^63,64^. In Conv3D, the convolution kernels moves in 3 directions, the input and output data of Conv3D is 4-dimensional^65^. Conv3D is mainly used for 3D image data, for instance, magnetic resonance imaging (MRI) data. MRI data is widely used to examine the brain, spinal cord, internal organs, etc., computer tomography (CT) scans are also three-dimensional data, which is an example of the creation of a series of X-ray images taken from different angles around the body. Conv3D are used to classify the medical data or extract features from it^66–68^. Figure 2 shows a one-dimensional (1D) convolution operation, where ${x}_{1} \; to \; {x}_{6}$ represent the inputs while ${c}_{1} \; to \; {c}_{4}$ represent the feature maps after 1D convolution. The red, blue, and green connections are the links between the input layer and the convoluting layer and each connection is weighted while connections that have the same color have equivalent weight value. Thus, only 3 weight values are needed in Fig. 2 to implement the convolution operation. One major advantage of the CNN model lies in its easy training phase due to the fewer number of weights compared to the number of weights in a fully-connected architecture. Furthermore, it allows the effective extraction of important features. Each convolutional layer may be represented as follow^69^:

1 $${h}_{ij}^{k}=f({({W}^{k}*x)}_{ij}+{b}_{k})$$

where $f$ is the activation function, ${W}^{k}$ is weights of the kernel linked to the *k*th feature map, while $*$ represents a convolution operator.

The considered CNN in this study has a fully connected layer and three convolutional layers; the selection of the convolutional layer channels was based on grid search. Furthermore, the activation function used is the rectified linear units (ReLU) while adaptive moment estimation (Adam) is used as the optimization algorithm. The ReLU can be expressed thus:

2 $$f\left(x\right)=\mathrm{max}(0,x)$$

The one-dimensional (1D) convolution operator is used to ensure simplification of the modeling processes, as well as to ensure real-time *Q*_*flow*_ prediction. The 1D convolution operator can make a direct prediction of the 1D *Q*_*flow*_ data.

### Long short-term memory

**Figure 3:**
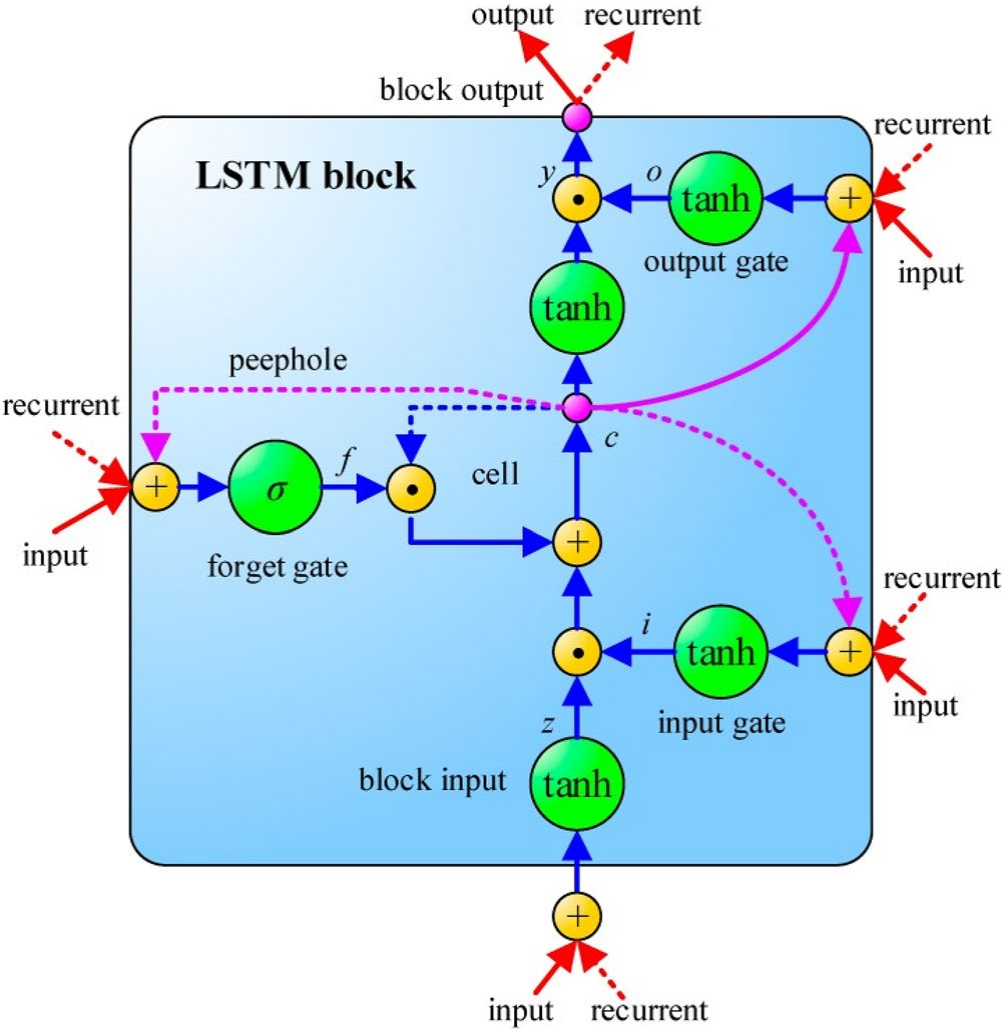
Topological structure of Long Short-Term Memory (LSTM) Network used in this study for the prediction of short-term (hourly) streamflow (*Q*, m^3^ s^−1^) at Brisbane River and Teewah Creek. Symbols as per “Theoretical overview” section.

Recurrent neural network (RNN) are powerful and robust type of artificial neural networks that uses existing time-series data to predict the future data over a specified length of time^70^. However, the RNNs can only recollect the recent information but not the earlier information^56^. Though the RNNs can be trained by back-propagation, it will be very difficult to train them for long input sequences due to vanishing gradients. Hence, the main drawback of the RNN architecture is its shorter memory to remember the features, vanishing and exploding gradients^71,72^. In order to overcome the vanishing and exploding gradients problem LSTM model was proposed^73^, LSTMs are a special class of RNN that relies on special units called memory blocks in their hidden layers; these memory blocks perform the role of the normal neurons in the hidden layers^73,74^. There are also three gate units in the memory blocks called input, output, and forget gates; these gates help in updating and controlling the flow of information through the memory blocks^75^. The LSTM network is calculated as follows^76^: (i) if the input gate is activated, any new input information into the system will be accumulated to the cell; (ii) the status of the previous cell is forgotten if the forget gate is activated; (iii) the propagation of the output of the latest cell to the ultimate state is controlled by the output gate. Figure 3 depicts the LSTM architecture.

Regarding streamflow prediction, the historical lagged input data is represented as $x=({x}_{1},{x}_{2},\dots ,{x}_{T})$ while the predicted data is depicted as $y=({y}_{1},{y}_{2},\dots ,{y}_{T})$. The computation of the predicted streamflow series is performed thus^77^:

3 $$Input\text{ gate }{i}_{t}=\sigma ({W}_{ix}{x}_{t}+{W}_{im}{m}_{t-1}+{W}_{ic}{c}_{t-1}+{b}_{i})$$

$$Forget\text{ gate }{f}_{t}=\sigma ({W}_{fx}{x}_{t}+{W}_{fm}{m}_{t-1}+{W}_{fc}{c}_{t-1}+{b}_{f})$$

$$\text{Output gate }{o}_{t}=\sigma ({W}_{ox}{x}_{t}+{W}_{om}{m}_{t-1}+{W}_{oc}{c}_{t}+{b}_{o})$$

$${c}_{t}={f}_{t}\circ {c}_{t-1}+{i}_{t}\circ g({W}_{cx}{x}_{t}+{W}_{cm}{m}_{t-1}+{b}_{c})$$

$${m}_{t}={o}_{t}\circ h({c}_{t})$$

$${y}_{t}={W}_{ym}{m}_{t}+{b}_{y}$$

where ${c}_{t}$: the activation vectors for cell, ${m}_{t}$: activation vectors for each memory block, $W$: weight, $b$: bias vectors, $\circ$: scalar product, $\sigma (.)$: gate activation function, $g(.)$: input activation function, $h(.)$: output activation function.

### Proposed deep CNN-LSTM network

**Figure 4:**
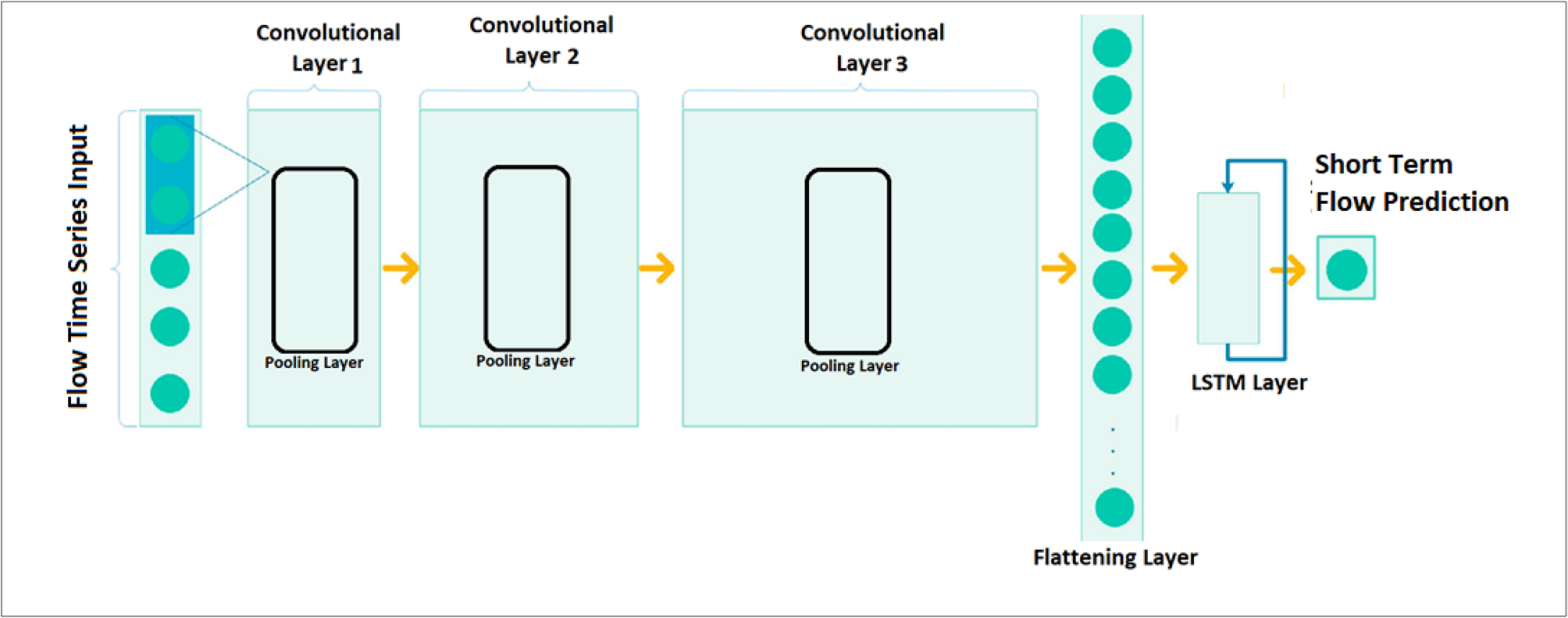
Topological structure of Convolutional neural Network (CNN) integrated with Long Short-Term Memory (LSTM) Network used in this study for the prediction of short-term (hourly) streamflow (*Q*, m^3^ s^−1^) at Brisbane River and Teewah Creek.

Figure 4 shows the proposed CNN-LSTM architecture in which the lagged hourly streamflow series serve as the inputs while the next hour streamflow is the output. In the proposed CNN-LSTM architecture, the first half is CNN that is used for feature extraction while the latter half is LSTM prediction that is for the analysis of the CNN-extracted features and for next-point streamflow prediction. There are three ID convolution layers in the CNN part of the proposed CNN-LSTM architecture.

### Deep neural network

There is a close similarity between the DNN concept and artificial neural network with many hidden layers and nodes in each layer. It can be trained on a set of features which will be later used for the objective function approximation^78^. The naming of DNNs is based on the networks as they are typically a compilation of numerous functions. The notable application of DNN is the prediction of global solar radiation and wind speed^79–81^.

## Study area and model development

**Figure 5:**
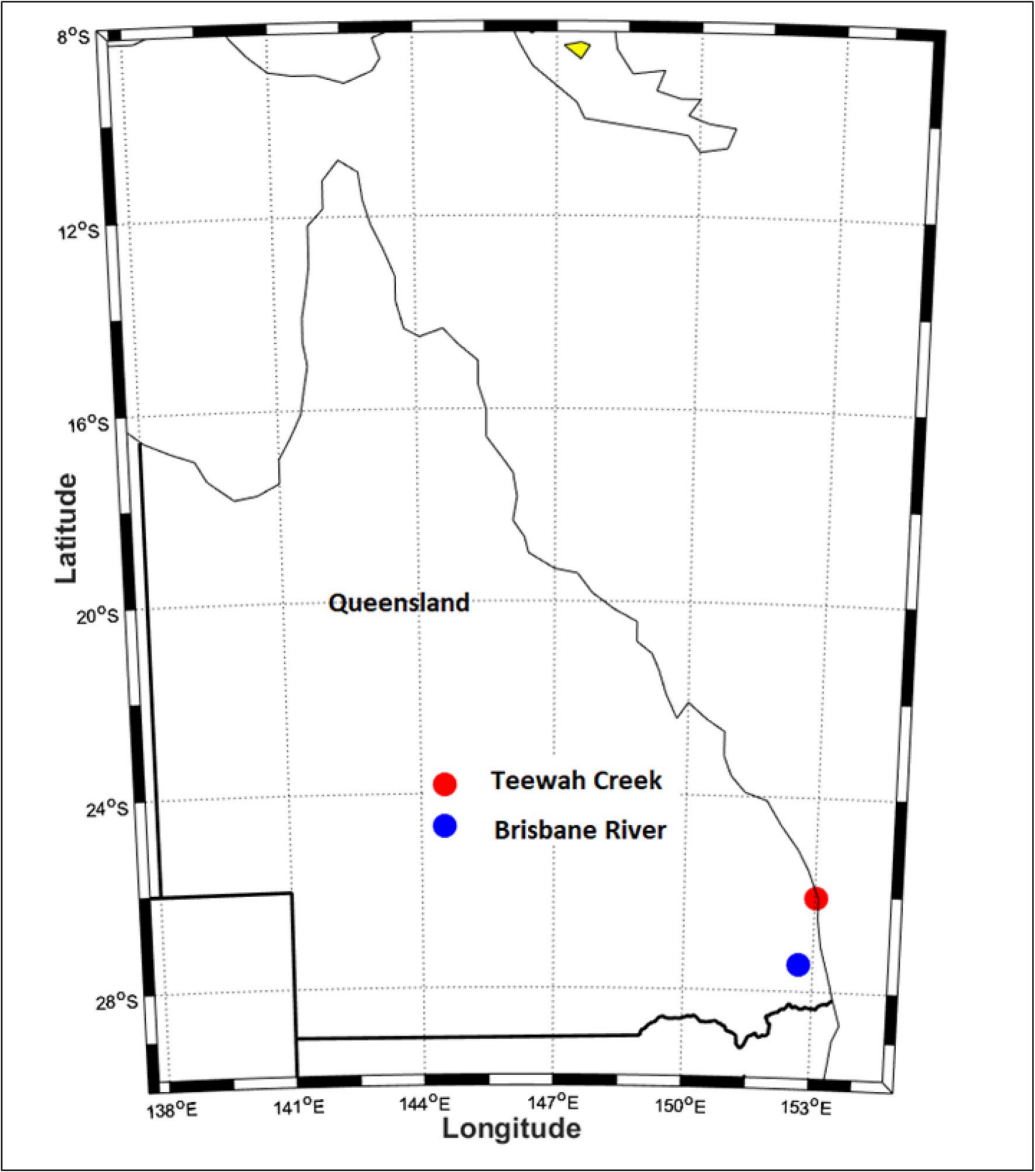
Location of Brisbane River and Teewah Creek study site in Australia, where experiments are carried out to validate the Deep Learning model for the prediction of hourly streamflow (*Q*, m^3^ s^−1^).

**Figure 6:**
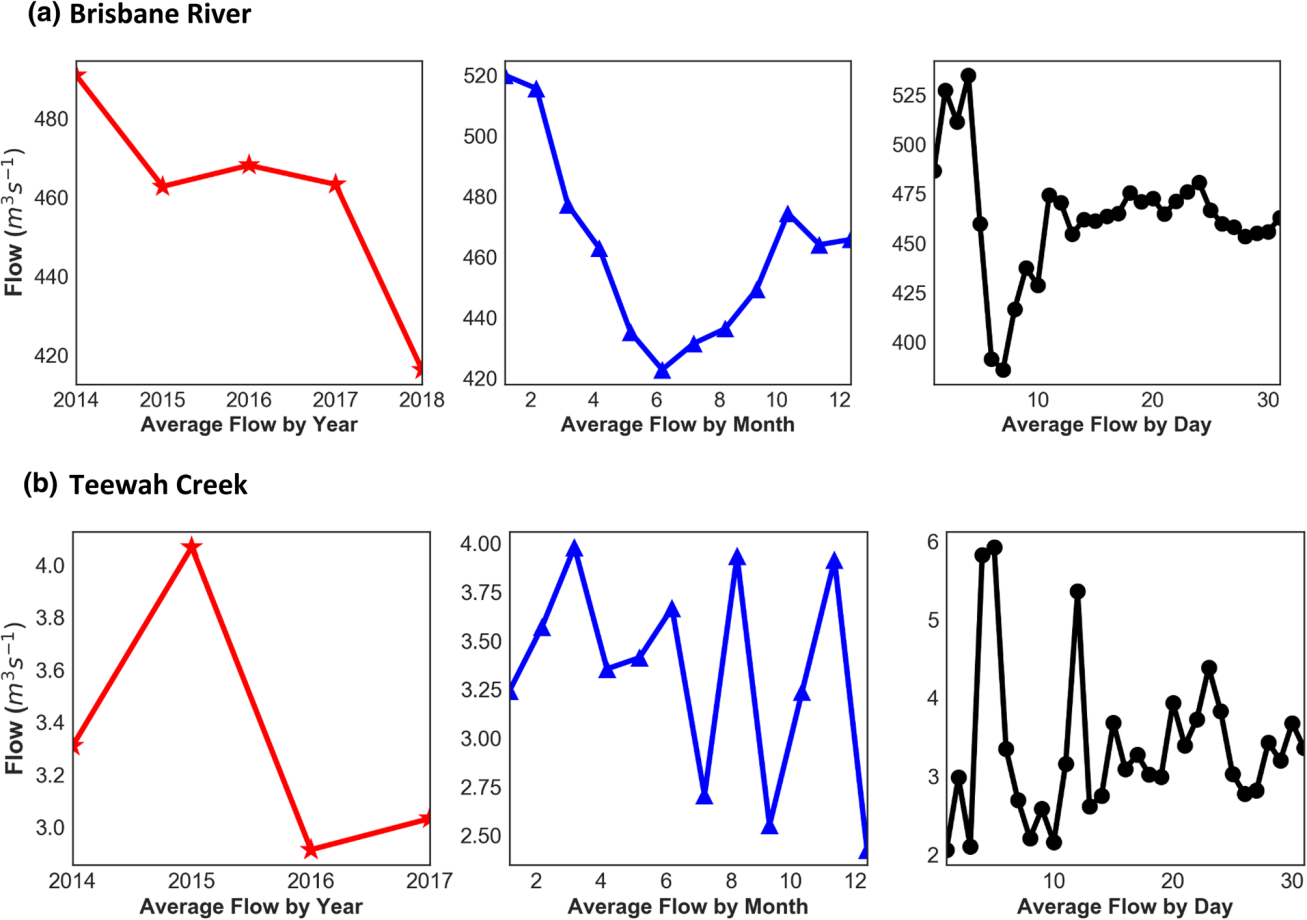
Variation of streamflow (*Q*, m^3^ s^−1^) by year, month and day for (**a**) Brisbane River and (**b**) Teewah Creek.

**Figure 7:**
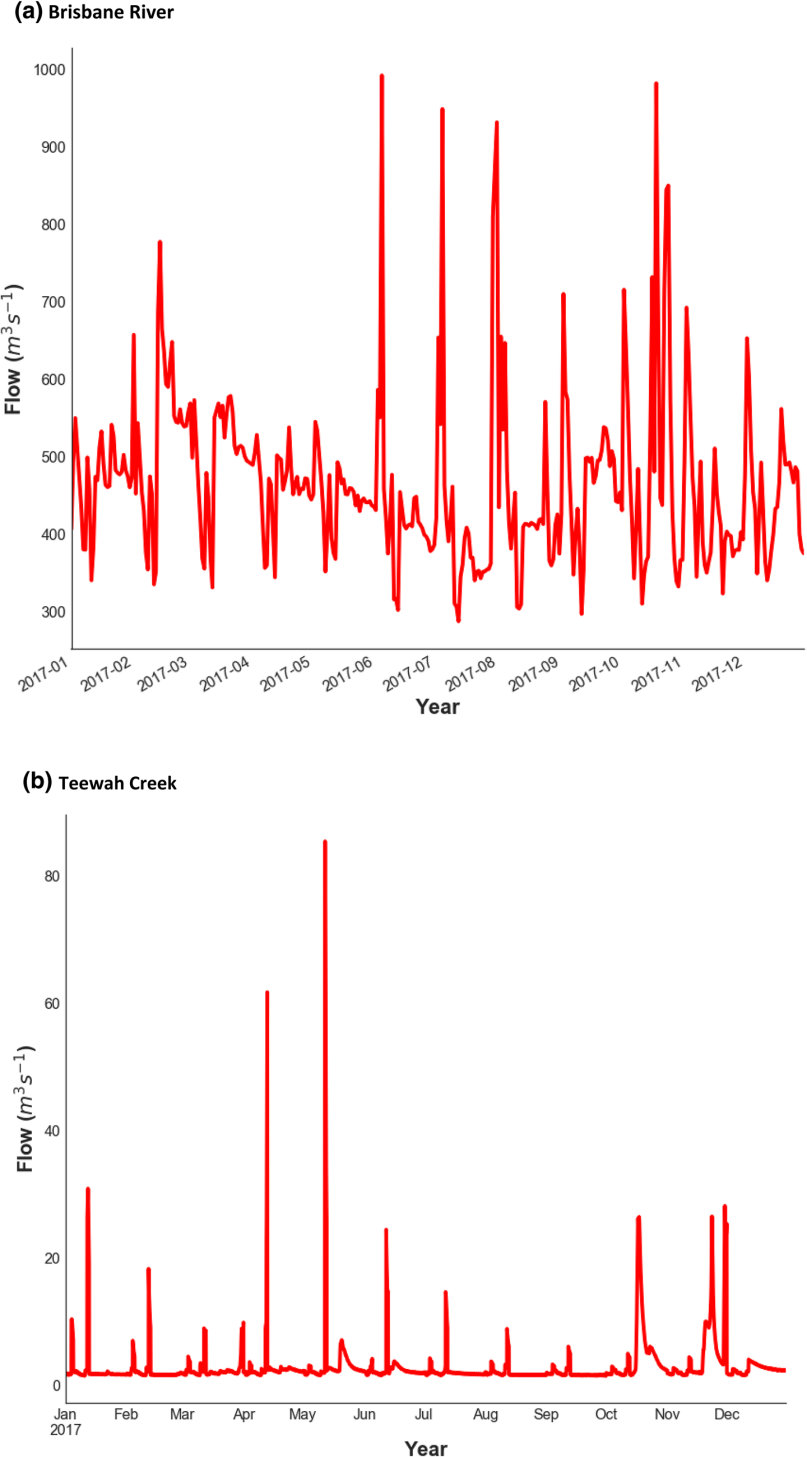
Hydrograph of streamflow during 2017 for (**a**) Brisbane River and (**b**) Teewah Creek, where the current study being done for hourly streamflow (*Q*, m^3^ s^−1^) prediction.

In order to develop a prediction model based on deep learning, conventional AI and ensemble models, this study has utilized lagged hourly data of streamflow (*Q*_*flow*_) from 01-January-2014 to 16-October-2017. Figure 5 plots a geographic map of the present study site, namely Brisbane River (Brisbane) and Teewah Creek (Noosa). The hourly streamflow (*Q*_*flow*_) data were acquired from the Water Monitoring Data Portal (Dept of Environment & Resource Management, http://watermonitoring.dnrm.qld.gov.au/host.htm). Figure 6 plots an average *Q*_*flow*_ characteristics for Brisbane River and Teewah Creek by year, month, and day. It can be seen from the figure that the *Q*_*flow*_ of the Brisbane river is more than that of Teewah creek, for Brisbane River the *Q*_*flow*_ is minimum at June whereas for Teewah creek *Q*_*flow*_ is significantly reduced at July, September and December. Similarly, the peak *Q*_*flow*_ occurs in February for Brisbane river whereas for Teewah creek the peak *Q*_*flow*_ occurs at March, June, August, and November. In addition, the time series plot of the *Q*_*flow*_ for the year 2017 is shown in Fig. 7.

### Model development

#### Data preparation

**Figure 8:**
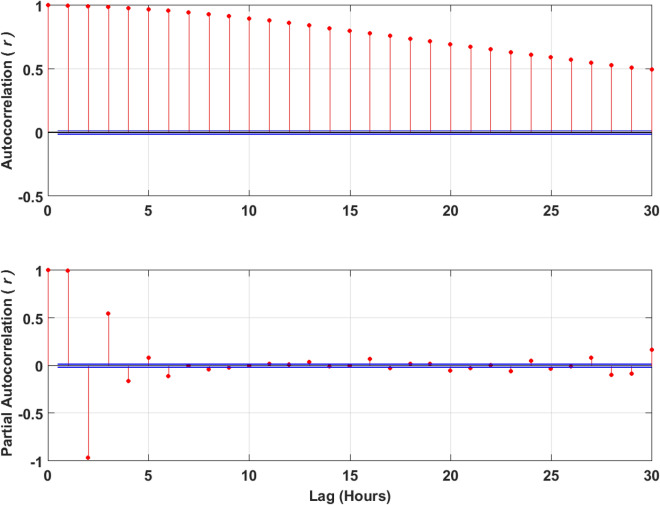
Autocorrelation (ACF) and partial autocorrelation (PACF) plot of the streamflow (*Q*, m^3^ s^−1^) time series for Brisbane river with lag in hours in order to make the input matrix of lagged streamflow series for the model input.

During data preparation, the first step is to determine the stationarity of the *Q*_*flow*_ time series. To do this, the Dicky–Fuller (DF) test was used in this study. With the application of the DF test, it implies that the null-hypothesis which suggests that the *Q*_*flow*_ time series is non-stationary, will be rejected. The next step is correlation analysis phase which aims at identifying the model order. The autocorrelation function (ACF) analysis was adopted in this study for the determination of the input of the *Q*_*flow*_ prediction model; this implies the determination of the input values that correlates maximally with the prediction values (Fig. 8). The major aim of using the ACF analysis method is to perform prediction tasks^82^. Owing to the stationarity of the *Q*_*flow*_ time series data, the computed 1-h interval autocorrelation function deteriorates at values < 0.27 as shown in Fig. 8 (the so-called correlation time (τc) in about 6-h (i.e., 6 lags of 1-h)). *Q*_*flow(t)*_ is considered the time series variable while the vector (*Q*_*flow(t-6)*_*, Q*_*flow(t-5)*_*, Q*_*flow(t-4)*_*, Q*_*flow(t-3)*_*, Q*_*flow(t-2)*_*, Q*_*flow(t-1)*_*, Q*_*flow(t)*_) is used in the next step as the input for the prediction of the value *Q*_*flow(t*+*1)*_.

#### Data normalization

Using Eq. (5), the modelled dataset was scaled between 0 and 1 to avoid the high values of variation in the dataset for easier simulation and converted to its original scale after modeling using Eq. (6)^83,84^, where *Q*_*flow*_, *Q*_*flow(min)*_ and *Q*_*flow(max*)_ represent the input data value and its overall minimum and maximum values, respectively

4 $${Q}_{flow(n)}=\frac{{{Q}_{flow(}}_{actual)}-{{Q}_{flow(}}_{\mathit{min})}}{{{Q}_{flow(}}_{\mathit{max})}-{{Q}_{flow(}}_{\mathit{min})}}$$

5 $${{Q}_{flow(}}_{actual)}={Q}_{flow(n)}\left({{Q}_{flow(}}_{\mathit{max})}-{{Q}_{flow(}}_{\mathit{min})}\right)\hspace{0.25em}+{{Q}_{flow(}}_{\mathit{min})}$$

After normalization the data are segregated into training and testing sets as demonstrated in Table 1. The *Q*_*flow*_ prediction was done for the year 2018 in the different range, spanning from 1-Week to 9-Months.

**Table 1:** Data segregation in terms of training, validation and testing for the hourly streamflow (*Q*, m^3^ s^−1^) prediction at Brisbane River and Teewah Creek.

Dataset	Training			Validation	Testing			
	Period	Data point	Interval (h)	Percentage of train data	Period	Data point	Interval (h)	Percentage
1W prediction	01-Jan-14 to 09-Oct-2017	41,812	1	10	10-Oct-2017 to 16-Oct-2018	167	1	0.4
2W prediction	01-Jan-14 to 01-Jan-2018	41,619	1	10	02-Jan-2018 to 16-Oct-2018	360	1	0.9
4W prediction	01-Jan-14 to 16-Sept-2018	41,258	1	10	31-Jan-2017 to 16-Oct-2018	721	1	1.7
20% of total data prediction	01-Jan-14 to 30-Jan-2017	33,589	1	10	31-Jan-2017 to 16-Oct-2018	8390	1	20.0

### Main model development

This study developed a 3-layer CNN model, 6-layer LSTM model, 4-layer CNN-LSTM model, and 4-layer DNN model. Table 2 presents the hyperparameters of the respective models which are selected based on the trial-and-error method as presented in Table 3. Some of these hyperparameters are model specific.

**Table 2:** The architecture of Deep learning models (Convolutional Neural Network (CNN) and CNNLSTM) and the respective conventional data-driven (MLP, ELM) and ensemble model (DT, GBM, XGB, MARS) used in the corresponding model development for Brisbane River and Teewah Creek streamflow (*Q*, m^3^ s^−1^) prediction, also the averaged training time for the optimum model is shown.

Model	Model hyperparameters	Hyperparameter selection	Run time (MM: SS)
CNNLSTM	Filter1	[50, **80**,100,200]	**20:42**
	Filter 2	[40,**50**,60,70,80]	
	Filter 3	[20,**10**,30,5]	
	LSTM cell units	[40,50,60,**100,**150]	
	Epochs	[1000,1200,**300**,400,700]	
	Batch size	[400,**500**,800,1000,750]	
CNN	Filter1	[**50**, 60,100,200]	**16:18**
	Filter 2	[**40**,50,60,70,130]	
	Filter 3	[20,10,30,**5**]	
	Epochs	[1000,1200,**300**,400,700]	
	Batch size	[**400**,500,800,1000,750]	
LSTM	LSTM cell 1	[50, 60,**100**,200]	**14.47**
	LSTM cell 2	[**40**,50,60,70,130]	
	LSTM cell 3	[20,10,30,**5**]	
	LSTM cell 4,5 and 6	[Fixed as 30,20,10]	
	Epochs	[1000,1200,**300**,400,700]	
	Drop rate	[0.1,0.2]	
	Batch size	[**400**,500,800,1000,750]	
DNN	Hiddenneuron 1	[100,200,300,400,**50**]	**10:18**
	Hiddenneuron 2	[20,**30**,40,50,60,70]	
	Hiddenneuron 3	[10,**20**,30,40,50]	
	Hiddenneuron 4	[5,6,7,8,**12**,15,18]	
	Epochs	[**1000**,1200,1500,1800,2000]	
	Batch size	[800,1000,**1200**,1500,1700,400]	
MLP	Activation	[**ReLU**, logistic, tanh]	**7:12**
	Solver	[**Adam**, lbfgs]	
	Learning rate	['constant', '**invscaling**', 'adaptive']	
	Maximum iteration	[500,1000,1500,2000]	
	Hidden layer size	[(100,), (150,), **(50,)**, (200,), (40,), (75,)]	
ELM	Hiddenneuron 3	[20,30,**40**,50]	**4:25**
	Activation function	[ReLU, logistic, **tanh**]	
GBM and XGB	Learning rate	[0.01, 0.1 ,**0.001**, 0.005]	**9:08**
	Maximum depth of the individual regression estimators	[5,8,**10**,20,25]	
	Number of boosting stages to perform	[50,**100**,150,200]	
	Minimum number of samples to split an internal node	[20]	
	Number of features for best split	['**auto**', 'sqrt', 'log2']	
MARS	Maximum term generated by forward pass	[10,**20**,30]	**7:16**
	Maximum degree of terms generated by forward pass	[5,**10**,15,20]	
Decision Tree	Maximum depth of the tree	[5,**10**,20]	**3:30**
	Minimum number of samples to split an internal node	[20]	
	Number of features for best split	['**auto**', 'sqrt', 'log2']	

ReLU, Adam and lbfgs stands for Rectified Linear Units, adaptive moment estimation and limited memory Broyden–Fletcher–Goldfarb–Shanno algorithm respectively. The selected parameter for the prediction of streamflow are bold faced.

**Table 3:** The architecture of Deep learning (DL) models (Convolutional Neural Network (CNN), Long Short Term Memory Network (LSTM), Deep Neural Network (DNN), and the respective backpropagation algorithm used in the DL model development for Brisbane River and Teewah Creek streamflow (*Q*, m^3^ s^−1^) prediction in hourly basis.

Architecture of deep learning											
Model	Layer 1 (L1)	L1 activation function	Dropout percentage	Layer 2 (L2)	L2 activation function	Layer 3 (L3)	Layer 4,5 and 6 (L4, L5 and L6)	L4, L5 and L6 activation function	Batch size	Epochs	
LSTM	100	ReLU	0.1	40	ReLU	5	30,20 and 10	ReLU	400	300	
DNN	50	ReLU	0.1	30	ReLU	20	12	ReLU	1200	1000	

	Convolution layers 1 (C1)	Convolution layers 2 (C2)	Convolutional layers 3 (C3)	Activation function	Pooling size	Padding	LSTM layer (L1)	L1 Activation function	Dropout rate	Batch Size	Epochs
CNN-LSTM	80	50	10	ReLU	2	Same	100	ReLU	0.1	500	300
CNN	50	40	5	ReLU	2	Same				400	300

Architecture of backpropagation (BP) algorithm for deep learning											
BP optimizers for deep learning model	Alpha, α	Epsilon, e	Beta, b_1_	Beta, b_2_							
Adaptive moment estimation, (Adam)	0.001	1E−07	0.99	0.99							

ReLU stands for Rectified Linear Units.

α = Learning rate, the proportion that weights are updated, e = Is a very small number to prevent any division by zero in the model implementation, b_1_ = The exponential decay rate for the 1st moment estimates, b_2_ = The exponential decay rate for the 2nd moment estimates.

#### Common hyperparameters

The DL modes share the following four common hyperparameters:

- Activation function: All the network layers rely on the same activation function ReLU except for the output layer.
- Dropout: This was considered a potential regularization technique for minimizing the issue of overfitting in order to improve the training performance^84^. Hence, dropout selects a fraction of the neurons (defined as a real hyperparameter in the range of 0 and 1) at each iteration and prevent them from training. This fraction of neurons was maintained at 0.1 in this study.
- Two statistics regularizations including L1: least absolute deviation and L2: least square error was used together with dropout. The role of the L1 and L2 penalization parameters is to minimize the sum of the absolute differences and sum of the square of the differences between the predicted and the target *Q*_*flow*_ values, respectively. The addition of a regularization term to the loss is believed to encourage smooth network mappings in a DL network by penalizing large values of the parameters; this will reduce the level of nonlinearity that the network models.
- Early stopping: The problem of overfitting was further addressed by introducing the early stopping (ES) criteria from Kera’s work^85^; the mode was set to minimum while patience was set to 20. This is to ensure that the training will terminate when the validation loss has stopped decreasing for the number of patience-specified epochs.

#### CNN model hyperparameter

- Filter size: The size of the convolution operation filter.
- Number of convolutions: The number of convolutional layers in each CNN.
- Padding: This study utilized the same padding in order to ensure that the dimensions of input feature map and output feature map are the same.
- Pool-size: A pooling layer is used between each convolution layer to avoid overfitting; this pooling layer helps in decreasing the number of parameters and network complexity. This study utilized a pool-size of 2 between layer 1 and 2.

#### CNN-LSTM model development

The proposed CNN-LSTM in this study is comprised of three convolutional layers with pooling operations; the selection of the convolutional layers channels was based on grid search. In the architecture of the model, the outputs of the flattening layer serve as the inputs of the LSTM recurrent layer while the LSTM recurrent layer is directly linked to the final outputs. The inputs of networks are the lagged matrix of hourly *Q*_*flow*_. The input parameter is the hourly *Q*_*flow*_ while the CNN-LSTM hyperparameters are deduced via the trial and error method as presented in Tables 2 and 3.

### Benchmark models

Open source Python libraries such as Scikit-Learn, PyEarth^86,87^ and Keras deep learning library^85,88^ were used to implement the conventional AI (MLP, ELM) and ensemble models (Decision Tree [DT], Random Forest [RFR], Gradient Boosting Regression [GBM], Extreme Gradient Boosting [XGB] and Multivariate Adaptive Regression Splines [MARS]. The hyperparameters of the conventional AI models and ensemble models were deduced through the trial-and-error method which are outlined in Table 2.

All the simulations were performed in a computer with Intel core i7 @ 3.3 GHz and 16 GB of RAM memory. For the simulation of model Python^89^ programming language was used with deep learning library like Keras^90^ and TensorFlow^91^. Several other programming tools are also used, for instance MATLAB for plotting^92^, Minitab for statistical analysis^93^.

### Performance metrics

In this section, the statistical metrics used for the model’s evaluation were reported. Following several machine learning models for hydrological process simulation, the following statistical metrics were used, including Correlation Coefficient (*r*), root mean square error (*RMSE*), mean absolute error (*MAE*), relative absolute error (*RAE*), Integral normalized root squared error (*INRSE*), Nash–Sutcliffe coefficient (*E*_*NS*_), Willmott’s index (*WI*), normalised root mean square error (*NRMSE*) and Legate and McCabe’s index (*LM*)^94–98^. Several researches found during their study that *E*_*NS*_ and *RMSE* are the most commonly used reliable metrics for prediction problem^99^.

Additionally, for the comparison of different models, the promoting percentages of mean absolute error (*P*_*MAE*_) and promoting percentages of root mean square error (*P*_*RMSE*_) were computed. Furthermore, the absolute percentage bias (*APB*) and Kling Gupta efficiency (*KGE*) as key performance indicators for *Q*_*flow*_ prediction, were calculated as well^100^.

The formulations of the metrics are:

i. Correlation Coefficient (*r*):
  6 $$r={\left(\frac{{\sum }_{i=1}^{N}\left({{Q}_{flow}}^{m}-<{{Q}_{flow}}^{m}>\right)\left({{Q}_{flow}}^{p}-<{{Q}_{flow}}^{p}>\right)}{\sqrt{{\sum }_{i=1}^{N}{\left({{Q}_{flow}}^{m}-<{{Q}_{flow}}^{m}>\right)}^{2}}\sqrt{{\sum }_{i=1}^{N}{\left({{Q}_{flow}}^{p}-<{{Q}_{flow}}^{p}>\right)}^{2}}}\right)}^{2}$$
ii. Mean Absolute Error (*MAE, *m^3^ s^−1^):
  7 $$MAE=\left(\sum^{\underset{t=1}{N}}|{{Q}_{flow}}^{m}-{{Q}_{flow}}^{p}|\right)/N$$
iii. Relative Root Mean Square Error (*RMAE, %*):
  8 $$RMAE=\frac{1}{N}{\sum }_{i=1}^{N}\left|\frac{\left({{Q}_{flow}}^{m}-{{Q}_{flow}}^{p}\right)}{{{Q}_{flow}}^{m}}\right|\times 100$$
iv. Root Mean Square Error (*RMSE, *m^3^ s^−1^):
  9 $$RMSE=\sqrt{\frac{1}{N}{\sum }_{i=1}^{N}{\left({{Q}_{flow}}^{m}-{{Q}_{flow}}^{p}\right)}^{2}}$$
v. Absolute Percentage Bias (*APB, %*):
  10 $${\text{APB}}=\left[\frac{{\sum }_{i=1}^{N}({{Q}_{flow}}^{m}-{{Q}_{flow}}^{p})*100}{{\sum }_{i=1}^{N}{{Q}_{flow}}^{m}}\right]$$
vi. Kling Gupta Efficiency (*KGE*):
  11 $$KGE=1-\sqrt{(r-1{)}^{2}+{\left(\frac{<{{Q}_{flow}}^{p}{>}}{<{{Q}_{flow}}^{m}{>}}-1\right)}^{2}+{\left(\frac{C{V}_{p}}{C{V}_{s}}\right)}^{2}}$$
vii. Integral Normalized Mean Square Error (*INRSE*):
  12 $$INRSE=\sqrt{\frac{{\sum }_{i=1}^{N}({{Q}_{flow(i)}}^{m}-{{Q}_{flow(i)}}^{p}{)}^{2}}{{\sum }_{i}^{N}({{Q}_{flow(i)}}^{m}-<{{Q}_{flow(i)}}^{m}>{)}^{2}}}$$
viii. Normalized Root Mean Square Error (*NRMSE*):
  13 $$NRMSE=\frac{{\sum }_{i=1}^{N}({{Q}_{flow(i)}}^{m}-{{Q}_{flow(i)}}^{p}{)}^{2}}{{{Q}_{flow(i)}}^{m}}$$
ix. Relative absolute error (*RAE, %*):
  14 $$RAE=\frac{{\sum }_{i=1}^{N}\left|{{Q}_{flow}}^{m}-\left.{{Q}_{flow}}^{p}\right|\right.}{{\sum }_{i=1}^{N}\left|{{Q}_{flow}}^{m}-<{{Q}_{flow}}^{m}>\right|}*100$$
x. Promoting Percentages of Mean Absolute Error (*P*_*MAE*_):
  15 $${P}_{M{\text{AE}}}=|(MA{E}_{1}-MA{E}_{2})/MA{E}_{1}|$$
xi. Promoting Percentages of Root Mean Square Error (*P*_*RMSE*_)
  16 $${P}_{RM{\text{SE}}}=|(RMS{E}_{1}-RMS{E}_{2})/RMS{E}_{1}|$$
xii. Nash–Sutcliffe coefficient (*E*_*NS*_):
  17 $${E}_{NS}=1-\frac{{\sum }_{i=1}^{N}{\left[{{Q}_{flow}}^{m}-{{Q}_{flow}}^{p}\right]}^{2}}{{\sum }_{i=1}^{N}{\left[{{Q}_{flow}}^{m}-\langle {{Q}_{flow}}^{p}\rangle \right]}^{2}}$$
xiii. Willmott’s index (*WI*):
  18 $$WI=1-\frac{{\sum }_{i=1}^{N}{\left[{{Q}_{flow}}^{m}-{{Q}_{flow}}^{p}\right]}^{2}}{{\sum }_{i=1}^{N}{\left[|({{Q}_{flow}}^{p}-\langle {{Q}_{flow}}^{m}\rangle )|+|({{Q}_{flow}}^{m}-\langle {{Q}_{flow}}^{p}\rangle )|\right]}^{2}}$$
xiv. Legate and McCabe’s index (*LM*):
  19 $$LM=1-\frac{{\sum }_{i=1}^{N}|{{Q}_{flow}}^{m}-{{Q}_{flow}}^{p}|}{{\sum }_{i=1}^{N}|{{Q}_{flow}}^{m}-\langle {{Q}_{flow}}^{m}\rangle |}$$
  where *r*: correlation coefficient, *CV*: coefficient of variation, ${{Q}_{flow}}^{m}$: measured *Q*_*flow*_, ${{Q}_{flow}}^{p}$: predicted *Q*_*flow*_,$<{{Q}_{flow}}^{m}>$: average value of the *Q*_*flow*_^*m*^*,* $<{{Q}_{flow}}^{p}>$: average value of the *Q*_*flow*_^*p*^*, N*: number of the dataset, $MA{E}_{1}$ and $RMS{E}_{1}$: mean model performance metrics (CNN = LSTM), $MA{E}_{2}$ and $RMS{E}_{2}$: benchmarked model performance (CNN, LSTM, DNN, MLP, etc.).

## Applications results and analysis

In this section, the predictability performance of the proposed CNN-LSTM model and the comparable models for the four experimental tests are conducted for 1-Week, 2-Weeks, 4-Weeks, and 9-months [20% of total Streamflow data (Table 1)] for hourly *Q*_*flow*_ prediction at Brisbane River and Teewah Creek. Each experiment consists of 10 *Q*_*flow*_ prediction models, including the CNN-LSTM, CNN, LSTM, DNN, DT, ELM, GBM, MARS, MLP and XGB. The performance metrics of proposed CNN-LSTM, deep learning (CNN, LSTM, DNN), conventional AI and ensemble models in terms of *r, RMSE, MAE, WI, LM* and *E*_*NS*_ are shown in Tables 4 and 5. The model prediction results over the testing phase represent the ability of the predictive models in simulating the streamflow data. Thus, the following sections will be focused on the model evaluation and assessment over the testing phase.

**Table 4:** Comparison of CNNLSTM model performances with the comparative counterpart models: Convolutional Neural Network (CNN) and Long Short Term Memory Network (LSTM) as well as the Deep Neural Network (DNN), Multi-Layer Perceptron (MLP), Extreme Learning Machine (ELM), Gradient Boosting Regression (GBM), Decision Tree(DT), Multivariate Adaptive Regression Splines (MARS) and Extreme Gradient Boosting Regression (XGB) model as measured by the correlation coefficient (r), root mean square error (RMSE; m^3^ s^−1^) and mean absolute error (MAE; m^3^ s^−1^) in the testing phase for hourly streamflow (Q, m^3^ s^−1^) prediction.

Metrics	Sites	Prediction interval	CNN	CNN-LSTM	DNN	DT	ELM	GBM	LSTM	MARS	MLP	XGB
*r*	Brisbane River	20% data	0.856	**0.949**	0.888	0.508	0.803	0.344	0.927	0.643	0.728	0.307
		1-week	0.953	**0.980**	0.973	0.892	0.900	0.902	0.950	0.902	0.846	0.905
		2-week	0.999	**1.000**	**1.000**	0.982	0.999	0.996	0.999	0.982	0.979	0.996
		4-week	0.955	**0.985**	0.980	0.801	0.938	0.826	0.974	0.783	0.739	0.827
	Teewah Creek	20% data	0.997	**0.999**	0.996	0.980	0.997	0.985	0.999	0.997	0.994	0.985
		1-week	0.999	**1.000**	0.999	0.974	0.999	0.993	0.999	0.974	0.998	0.993
		2-week	0.999	**1.000**	**1.000**	0.982	0.999	0.996	0.999	0.982	0.979	0.996
		4-week	0.999	**1.000**	0.999	0.984	0.999	0.996	0.999	0.984	0.997	0.996
*RMSE* (m^3^s^−1^)	Brisbane River	20% data	2.641	**1.578**	2.328	11.222	3.091	5.644	1.879	4.166	3.638	5.799
		1-week	0.270	**0.176**	0.204	0.408	0.394	0.389	0.279	0.390	0.487	0.384
		2-week	0.402	**0.226**	0.290	1.776	0.377	0.866	0.296	1.776	1.918	0.870
		4-week	0.266	**0.155**	0.176	0.561	0.313	0.526	0.203	0.586	0.643	0.524
	Teewah Creek	20% data	0.373	**0.230**	0.443	1.055	0.405	0.925	0.265	0.417	0.587	0.906
		1-week	0.569	**0.318**	0.389	2.584	0.573	1.292	0.397	2.584	0.795	1.315
		2-week	0.402	**0.226**	0.290	1.776	0.377	0.866	0.296	1.776	1.918	0.870
		4-week	0.251	**0.176**	0.224	1.255	0.273	0.616	0.234	1.255	0.496	0.619
*MAE* (m^3^s^−1^)	Brisbane River	20% data	0.601	**0.150**	0.396	0.734	0.701	0.970	0.516	0.318	2.010	0.949
		1-week	0.225	**0.130**	0.152	0.275	0.312	0.321	0.238	0.222	0.428	0.318
		2-week	0.191	**0.112**	0.121	0.610	0.182	0.351	0.129	0.610	0.872	0.359
		4-week	0.203	**0.094**	0.128	0.394	0.239	0.440	0.161	0.406	0.508	0.437
	Teewah Creek	20% data	0.097	**0.054**	0.097	0.195	0.085	0.165	0.063	0.079	0.349	0.164
		1-week	0.361	**0.196**	0.231	1.263	0.371	0.713	0.235	1.263	0.475	0.742
		2-week	0.191	**0.112**	0.121	0.610	0.182	0.351	0.129	0.610	0.872	0.359
		4-week	0.101	**0.065**	0.079	0.329	0.105	0.190	0.079	0.329	0.262	0.198

Prediction was done for 1-hour horizon for 20% of total data(9-Months), 1-Week (1W), 2-Week (2W) and 4-Week (4W) Best Model is highlighted in boldfaced.

**Table 5:** Performance evaluation of CNN-LSTM model with the comparative counterpart models: Convolutional Neural Network (CNN) and Long Short Term Memory Network (LSTM) as well as the Deep Neural Network (DNN), Multi-Layer Perceptron (MLP), Extreme Learning Machine (ELM), Gradient Boosting Regression (GBM), Decision Tree(DT), Multivariate Adaptive Regression Splines (MARS) and Extreme Gradient Boosting Regression (XGB) model as measured by Willmott’s index (WI), ) Legates and McCabe’s Index (LM) and Nash–Sutcliffe Coefficient (E_NS_) in the testing phase for hourly streamflow (Q, m^3^ s^−1^) prediction.

Metrics	Sites	Prediction Interval	CNN	CNN-LSTM	DNN	DT	ELM	GBM	LSTM	MARS	MLP	XGB
*WI*	Brisbane River	20% data	0.966	**0.988**	0.975	0.716	0.950	0.888	0.982	0.920	0.931	0.880
		1-week	0.988	**0.995**	0.994	0.975	0.975	0.973	0.989	0.977	0.957	0.973
		2-week	1.000	**1.000**	1.000	0.996	1.000	0.999	1.000	0.996	0.994	0.999
		4-week	0.987	**0.996**	0.995	0.952	0.986	0.948	0.994	0.948	0.941	0.948
	Teewah Creek	20% data	0.999	**1.000**	0.999	0.995	0.999	0.996	1.000	0.999	0.998	0.996
		1-week	1.000	**1.000**	1.000	0.994	1.000	0.998	1.000	0.994	0.999	0.998
		2-week	1.000	**1.000**	1.000	0.996	1.000	0.999	1.000	0.996	0.994	0.999
		4-week	1.000	**1.000**	1.000	0.996	1.000	0.999	1.000	0.996	0.999	0.999
*LM*	Brisbane River	20% data	0.786	**0.952**	0.867	0.780	0.747	0.676	0.827	0.895	0.337	0.675
		1-week	0.762	**0.868**	0.854	0.738	0.684	0.649	0.777	0.788	0.509	0.648
		2-week	0.981	**0.989**	0.988	0.940	0.982	0.964	0.987	0.940	0.901	0.964
		4-week	0.779	**0.907**	0.879	0.642	0.795	0.505	0.854	0.635	0.578	0.510
	Teewah Creek	20% data	0.971	**0.984**	0.970	0.940	0.974	0.948	0.981	0.976	0.891	0.949
		1-week	0.975	**0.986**	0.984	0.913	0.974	0.950	0.984	0.913	0.967	0.948
		2-week	0.981	**0.989**	0.988	0.940	0.982	0.964	0.987	0.940	0.901	0.964
		4-week	0.983	**0.989**	0.986	0.944	0.982	0.967	0.987	0.944	0.954	0.965
E_NS_	Brisbane River	20% data	0.877	**0.955**	0.911	0.467	0.812	0.710	0.931	0.745	0.739	0.689
		1-week	0.949	**0.980**	0.975	0.907	0.900	0.886	0.958	0.914	0.813	0.886
		2-week	0.999	**1.000**	1.000	0.983	0.999	0.996	0.999	0.983	0.973	0.995
		4-week	0.944	**0.984**	0.981	0.826	0.950	0.763	0.976	0.816	0.799	0.764
	Teewah Creek	20% data	0.997	**0.999**	0.996	0.978	0.997	0.983	0.999	0.997	0.994	0.983
		1-week	0.999	**1.000**	0.999	0.976	0.999	0.993	0.999	0.976	0.998	0.993
		2-week	0.999	**1.000**	1.000	0.983	0.999	0.996	0.999	0.983	0.973	0.995
		4-week	0.999	**1.000**	0.999	0.985	0.999	0.996	0.999	0.985	0.997	0.996

Prediction was done for 1-hour horizon for 20% of total data (9-Months), 1-Week (1W), 2-Week (2W) and 4-Week (4W) Best Model is highlighted in boldfaced.

For both sites (Brisbane River and Teewah Creek), it can be seen that the $1.00\le r\ge 0.88$ for all deep learning model, $0.999\le r\ge 0.728$ for conventional AI and $0.344\le r\ge 0.996$ ensemble model for all prediction interval. Since *r* is parametric and oversensitive to extreme values^98^, the conclusion of model performance based on this coefficient is not sufficient. Therefore, further assessment of model performance was done using *MAE* and *RMSE.* With low *RMSE* (CNN-LSTM/$0.226\le RMSE\ge 0.155 \; \text{m}^{3}\;\text{s}^{-1} (Brisbane River))$ and *MAE* (CNN-LSTM/$0.196\le MAE\ge 0.054 \; \text{m}^{3}\; \text{s}^{-1} (Tewah Creek) )$ the CNN-LSTM model outperform the all conventional data driven [e.g. ELM/$0.182\le MAE\ge 0.701 \; \text{m}^{3} \text{s}^{-1}(Brisbane River)$] as well as the ensemble model [e.g. DT/$0.734\le MAE\ge 0.275 \; \text{m}^{3}\; \text{s}^{-1} (Teewah Creek) ]$ for all prediction interval of 1-Week, 2-Weeks, 4-Weeks and 9-Months (20% testing data).

Additionally, in hydrological model the *E*_*NS*_ is a widely used metric for prediction of streamflow, water level, drought etcetera and is considered as an expertise score calculated as the reasonable capability^100^ that presents the mean values of the *Q*_*flow*_. However, *E*_*NS*_ metric neglects the lower values and overestimates the larger ones^98^. In addition, Willmott's index (*WI*) metric is calculated due to its merits over the *r* and *E*_*NS*_. In the computation of the *WI* metric, errors and differences are given their appropriate weighting, which overcomes the insensitivity issues^98^. Further, *WI* and *E*_*NS*_ do not take the absolute value into account and are oversensitive to peak residual values^101^, therefore *LM* was taken into consideration for further model assessment. The *LM* is not overestimated since it takes absolute values into account^102^. As shown in Table 5, with high magnitude of *E*_*NS*_, *WI* and *LM*, CNN-LSTM model [1.00 $\le$ *WI* $\ge$ 0.96, 0.989 $\le$ *LM* $\ge$ 0.868, 1.00 $\le$ *E*_*NS*_ $\ge$ 0.955 (Brisbane River)] outperform all the models $[MLP: 0.994\le WI\ge 0.931, 0.901\le LM\ge 0.337, 0.973\le {E}_{Ns\cdot }\ge 0.739 \left(Brisbane River\right);DT: 0.952\le WI\ge 0.716, 0.982\le LM\ge 0.684, 0.983\le {E}_{Ns\cdot }\ge 0.467 \left(Brisbane River\right) ]$ for all the prediction levels for both sites.

**Figure 9:**
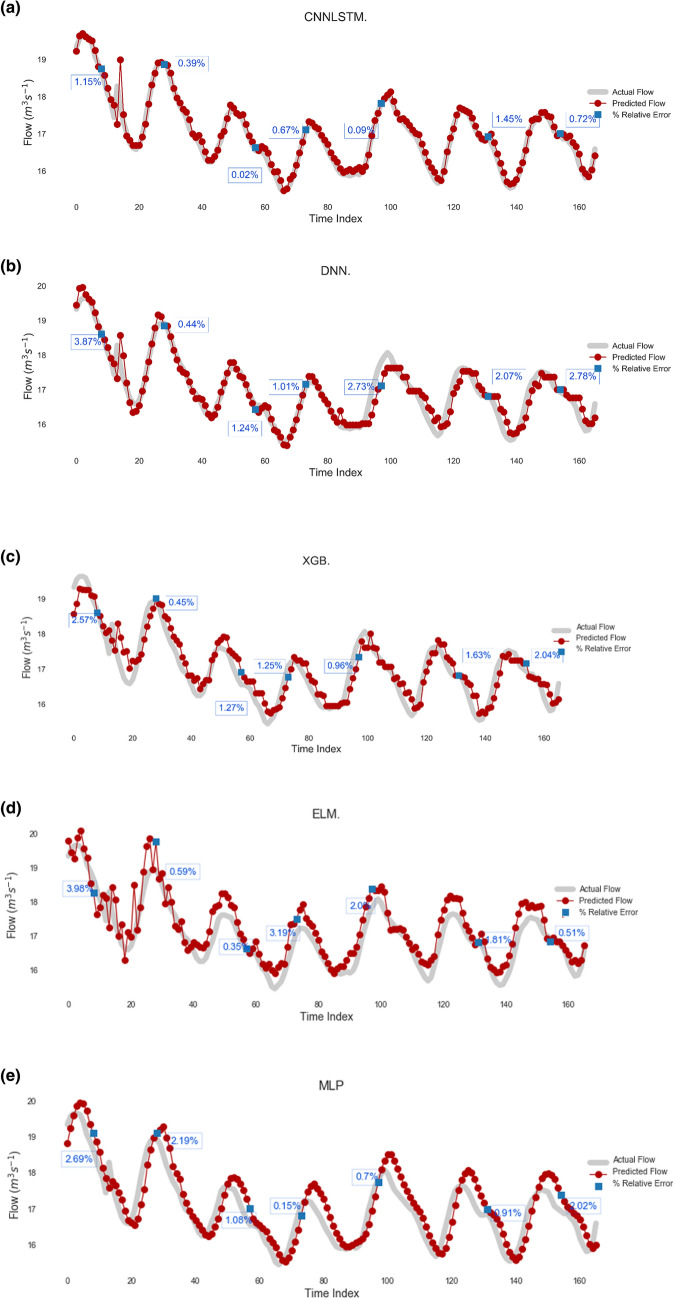
Hydrograph of predicted versus actual streamflow (*Q*, m^3^ s^−1^) from (**a**) CNN-LSTM model during test period (2-Weeks) compared with standalone model (**b**) Deep Neural Network (DNN), (**c**) Extreme Gradient Boosting Regression Model (XGB) , (**d**) Extreme Learning Machine (ELM) and (**e**) Multi-Layer Perceptron (MLP) for Brisbane River. The relative error are shown in blue color.

**Figure 10:**
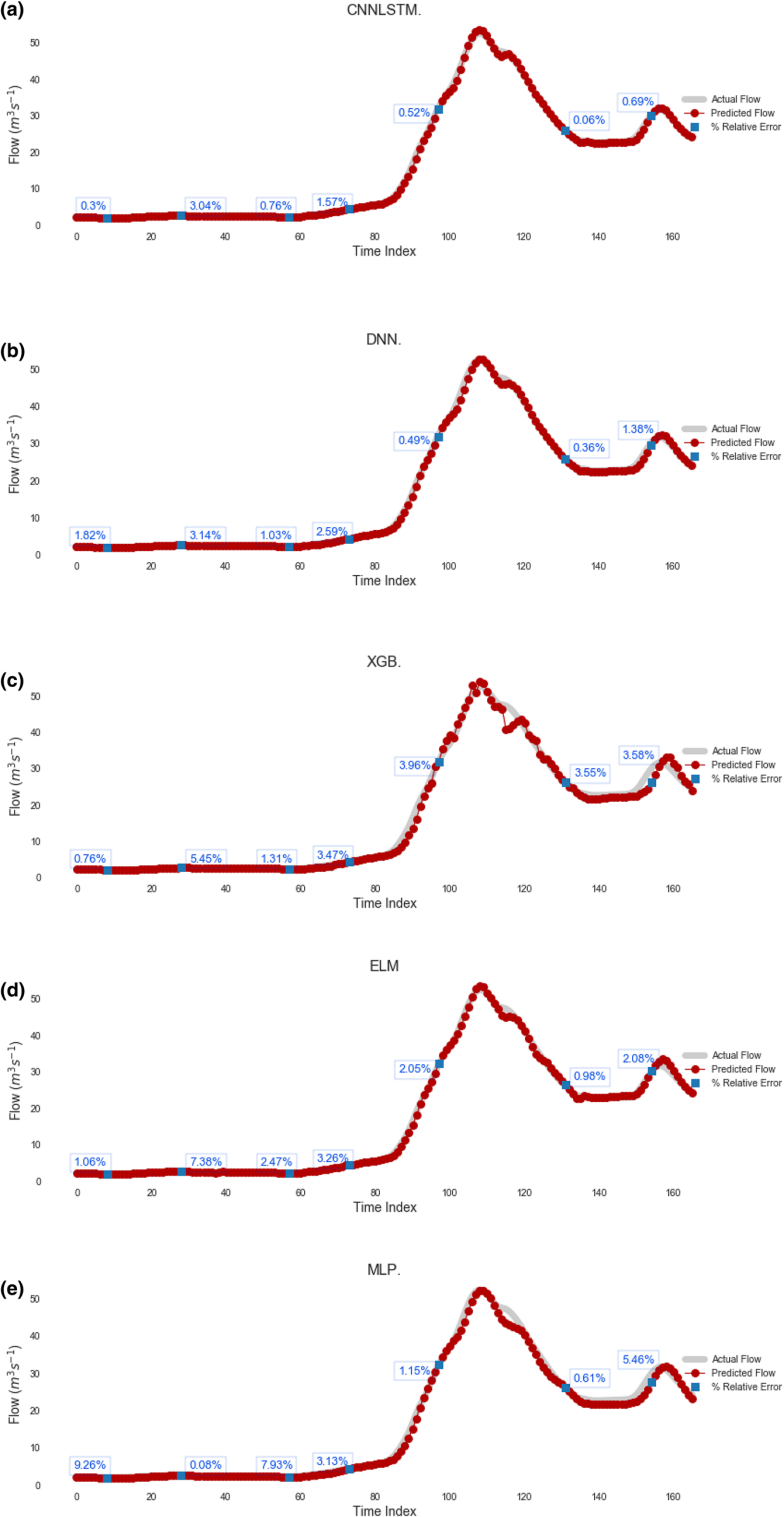
Hydrograph of predicted versus actual streamflow (Q, m^3^ s^−1^) from (**a**) CNN-LSTM model during test period (2 Weeks) compared with standalone model (**b**) Deep Neural Network (DNN), (**c**) Extreme Gradient Boosting Regression Model (XGB), (**d**) Extreme Learning Machine (ELM) and (**e**) Multi-Layer Perceptron (MLP) for Teewah Creek. The relative error are shown in blue color.

**Figure 11:**
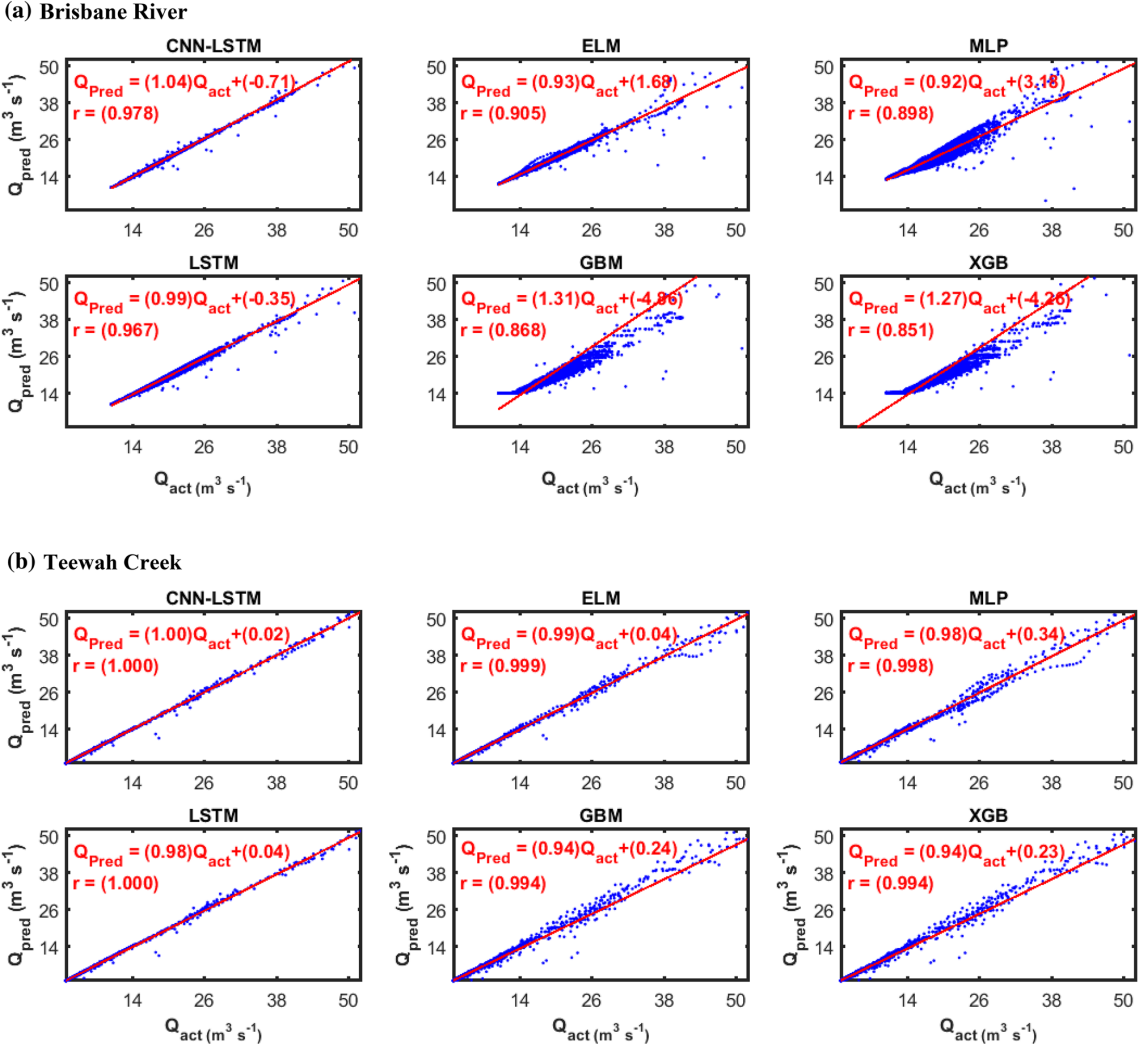
Scatterplot of predicted (*Q*_*pred*_) and actual (*Q*_*act*_) hourly streamflow (*Q*, m^3^ s^−1^) for (**a**) Brisbane River and (**b**) Teewah Creek using the CNN-LSTM, Extreme Learning Machine (ELM), Multi-Layer Perceptron (MLP), Long Short Term Memory Network (LSTM), Gradient Boosting Regression (GBM) and Extreme Gradient Boosting Regression Model (XGB) model. Least square regression equations of the form y = mx + C and the correlation coefficient (r) is inserted in each panel.

Figures 9 and 10 show the hydrograph and the scatterplots (Fig. 11) of both the actual and predicted *Q*_*flow*_ obtained by proposed CNN-LSTM model as well as conventional AI and ensemble models during the testing period. For the purpose of brevity, only the plots for prediction interval of 2-Weeks are shown. The hydrographs and the scatterplots demonstrate that the prediction of the CNN-LSTM model was closest to the observed *Q*_*flow*_ values in comparison to the other models. The fit line formula ($y=mx+c$) presented in scatterplots where *m* and *c* are the model coefficients, respectively, closer to the 1 and 0 with a higher *r* value of 1.00 than ELM, MLP, LSTM, GBM and XGB models. Additionally, in hydrograph the relative error (*RE*) percentage are also shown, indicating that the *RE* of the CNN-LSTM model is comparatively smaller than that of other comparable models.

It is worthwhile highlighting that ELM, MLP, XGB models are able to achieve a good predictability performance with the limitation in maintaining the good prediction for the high *Q*_*flow*_ values (Figs. 8 and 9). On the contrary, the CNN-LSTM model achieves a superior prediction result for the peak values compared to ELM, MLP and XGB models. The CNN-LSTM model only underestimates the peak values by 1.15% as opposed to 2.57%, 3.98% and 2.69% for the XGB, ELM and MLP, respectively for Brisbane River. This demonstrates the suitability of the CNN-LSTM for streamflow prediction.

To avoid the scale dependencies and impact of the outliers in the predicted streamflow, the *RAE*, *NRMSE* and *INRSE* were also recommended in some literatures^103^. Therefore, in this study further evaluation of model performance is conducted by using the *RAE, NRMSE* and *INRSE* (Table 6). For both sites, the CNN-LSTM model achieves a lower value of *RAE, NRMSE* and *INRSE*, outperforming the conventional AI and ensemble models. In line with the results presented in Tables 4 and 5, the integration of CNN and LSTM again has shown to enhance the prediction capability.

**Table 6:** Comparison of CNN-LSTM model performances with the comparative counterpart models: Convolutional Neural Network (CNN) and Long Short Term Memory Network (LSTM) as well as the Deep Neural Network (DNN), Multi-Layer Perceptron (MLP), Extreme Learning Machine (ELM), Gradient Boosting Regression (GBM), Decision Tree (DT), Multivariate Adaptive Regression Splines (MARS) and Extreme Gradient Boosting Regression (XGB) model, as measured by Normalized root mean squared error (NRMSE), relative absolute error (RAE) and Integral normalized root squared error (INRSE) in the testing phase.

Metrics	Sites	Prediction interval	CNN	CNN-LSTM	DNN	DT	ELM	GBM	LSTM	MARS	MLP	XGB
*NRMSE*	Brisabne River	20% data	0.011	**0.007**	0.010	0.047	0.013	0.024	0.008	0.017	0.015	0.024
		1-week	0.043	**0.028**	0.032	0.065	0.062	0.061	0.044	0.062	0.077	0.061
		2-week	0.008	**0.004**	0.006	0.035	0.007	0.017	0.006	0.035	0.038	0.017
		4-week	0.042	**0.024**	0.028	0.089	0.050	0.083	0.032	0.093	0.102	0.083
	Teewah Creek	20% data	0.004	**0.003**	0.005	0.012	0.005	0.011	0.003	0.005	0.007	0.011
		1-week	0.011	**0.006**	0.008	0.051	0.011	0.026	0.008	0.051	0.016	0.026
		2-week	0.008	**0.004**	0.006	0.035	0.007	0.017	0.006	0.035	0.038	0.017
		4-week	0.005	**0.003**	0.004	0.025	0.005	0.012	0.005	0.025	0.010	0.012
*RAE*	Brisabne River	20% data	0.199	**0.050**	0.132	0.244	0.233	0.322	0.172	0.106	0.668	0.315
		1-week	0.229	**0.132**	0.154	0.280	0.317	0.326	0.242	0.226	0.435	0.323
		2-week	0.019	**0.011**	0.012	0.061	0.018	0.035	0.013	0.061	0.087	0.036
		4-week	0.195	**0.090**	0.123	0.378	0.229	0.421	0.154	0.389	0.486	0.419
	Teewah Creek	20% data	0.030	**0.016**	0.030	0.059	0.026	0.050	0.019	0.024	0.106	0.050
		1-week	0.025	**0.014**	0.016	0.088	0.026	0.049	0.016	0.088	0.033	0.052
		2-week	0.019	**0.011**	0.012	0.061	0.018	0.035	0.013	0.061	0.087	0.036
		4-week	0.017	**0.011**	0.014	0.057	0.018	0.033	0.014	0.057	0.045	0.034
*INRSE*	Brisabne River	20% data	0.379	**0.226**	0.334	1.610	0.443	0.810	0.270	0.598	0.522	0.832
		1-week	0.217	**0.142**	0.164	0.328	0.317	0.313	0.224	0.314	0.392	0.309
		2-week	0.031	**0.017**	0.022	0.136	0.029	0.066	0.023	0.136	0.146	0.066
		4-week	0.211	**0.123**	0.140	0.446	0.249	0.418	0.161	0.466	0.511	0.416
	Teewah Creek	20% data	0.050	**0.031**	0.060	0.142	0.054	0.124	0.036	0.056	0.079	0.122
		1-week	0.036	**0.020**	0.024	0.161	0.036	0.081	0.025	0.161	0.050	0.082
		2-week	0.031	**0.017**	0.022	0.136	0.029	0.066	0.023	0.136	0.146	0.066
		4-week	0.025	**0.018**	0.023	0.127	0.028	0.062	0.024	0.127	0.050	0.063

Prediction was done for 1-h horizon for 20% of total data (9-Months), 1-Week (1W), 2-Week (2W) and 4-Week (4W) Best Model is highlighted in boldfaced.

Furthermore, a comparison of the CNN-LSTM model without other models is performed in terms of the *APB* and *KGE*. The *KGE* and *APB* evaluation for the prediction of hourly *Q*_*flow*_ reveals that the CNN-LSTM is the best performing model with *KGE* ≥ *0.991*, *APB* ≤ *0.527* and *KGE* ≥ *0.991, APB* ≤ *1.159* for Brisbane River and Teewah creek, respectively (Table 7), indicating a good model performance^104^ and making the CNN-LSTM model a reliable and powerful tool for the prediction of *Q*_*flow*_.

**Table 7:** Comparison of CNNLSTM model performances with the comparative counterpart models: Convolutional Neural Network (CNN) and Long Short Term Memory Network (LSTM) as well as the Deep Neural Network (DNN), Multi-Layer Perceptron (MLP), Extreme Learning Machine (ELM), Gradient Boosting Regression (GBM), Decision Tree(DT), Multivariate Adaptive Regression Splines (MARS) and Extreme Gradient Boosting Regression (XGB) model, as measured by Kling Gupta Efficiency (KGE) and the absolute percentage bias (APB) in the testing phase.

Metrics	Sites	Prediction interval	CNN	CNNLSTM	DNN	DT	ELM	GBM	LSTM	MARS	MLP	XGB
*APB*	Brisbane River	20% data	3.381	**0.856**	2.243	4.124	3.931	5.406	3.037	1.816	10.466	5.302
		1-week	1.279	**0.739**	0.865	1.568	1.795	1.826	1.348	1.268	2.425	1.807
		2-week	2.173	**1.284**	1.393	6.928	2.097	4.086	1.498	6.928	11.081	4.177
		4-week	1.151	**0.527**	0.723	2.218	1.344	2.473	0.908	2.274	2.882	2.456
	Teewah Creek	20% data	2.083	**1.159**	2.098	4.234	1.832	3.600	1.373	1.706	7.146	3.582
		1-week	2.231	**1.192**	1.410	7.655	2.274	4.403	1.429	7.655	2.885	4.588
		2-week	2.173	**1.284**	1.393	6.928	2.097	4.086	1.498	6.928	11.081	4.177
		4-week	1.937	**1.235**	1.510	6.204	1.985	3.649	1.484	6.204	5.215	3.804
*KGE*	Brisbane River	20% data	0.900	0.934	0.886	0.395	0.901	0.639	**0.950**	0.793	0.861	0.637
		1-week	0.959	**0.990**	0.952	0.916	0.956	0.906	0.917	0.921	0.874	0.896
		2-week	0.981	0.991	**0.993**	0.963	0.992	0.982	0.984	0.963	0.840	0.982
		4-week	0.878	**0.967**	**0.976**	0.890	0.897	0.812	0.954	0.879	0.841	0.813
	Teewah Creek	20% data	0.994	**0.998**	0.995	0.946	0.989	0.935	0.981	0.996	0.947	0.936
		1-week	0.979	**0.996**	0.994	0.960	0.990	0.982	0.992	0.960	0.996	0.981
		2-week	0.981	0.991	**0.993**	0.963	0.992	0.982	0.984	0.963	0.840	0.982
		4-week	0.986	**0.998**	0.987	0.970	0.984	0.985	0.982	0.994	0.947	0.983

Prediction was done for 1-h horizon for 20% of total data (9-Months), 1-Week (1W), 2-Week (2W) and 4-Week (4W) Best Model is highlighted in boldfaced.

**Figure 12:**
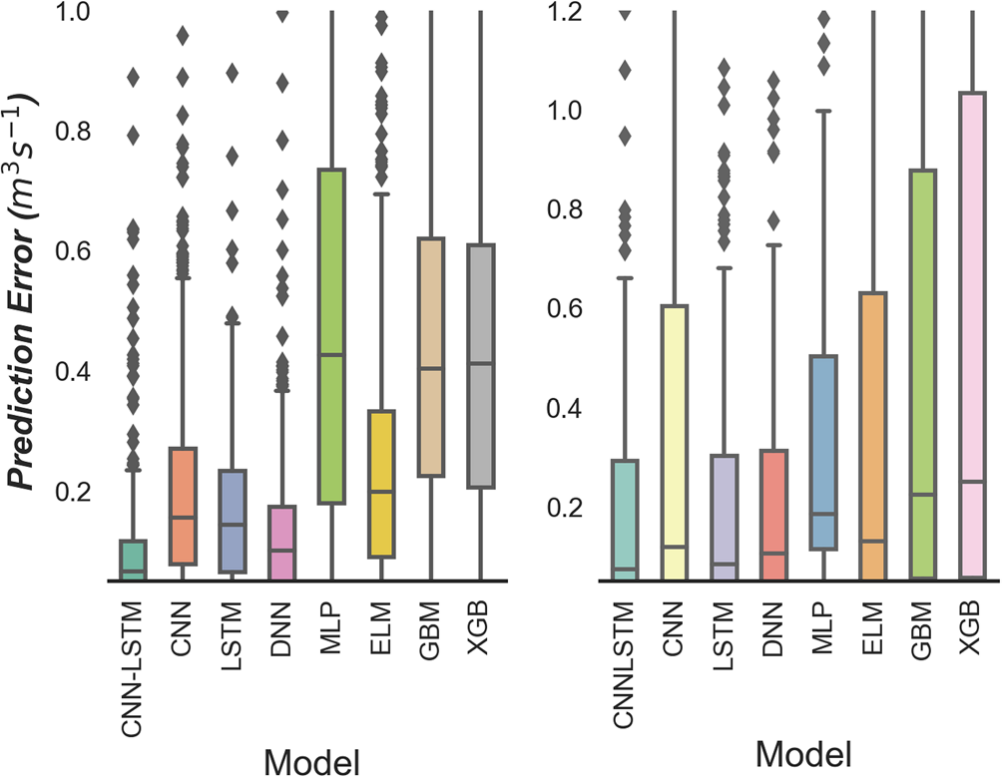
Box plots of spread of prediction error (*PE*, m^3^ s^−1^) for proposed CNN-LSTM model during test period compared with standalone model Convolutional Neural Network (CNN) and Long Short Term Memory Network (LSTM) as well as the Deep Neural Network (DNN), Multi-Layer Perceptron (MLP), Extreme Learning Machine (ELM), Gradient Boosting Regression (GBM) and Extreme Gradient Boosting Regression (XGB) model.

Figure 12 compares the boxplot of the proposed CNN-LSTM model with that of the standalone deep learning model as well as conventional AI and ensemble models. The ♦ markers in the figure demonstrate the outliers of the absolute prediction error *(|PE|)* of the testing data together with their upper quartile, median, and lower quartile. The distributions of the *|PE|* error acquired by the proposed CNN-LSTM model for all sites exhibit a much smaller quartile followed by the standalone deep learning models. By analysing Fig. 11, the accuracy of the proposed CNN-LSTM model for all sites is shown to be better than the comparative models.

**Figure 13:**
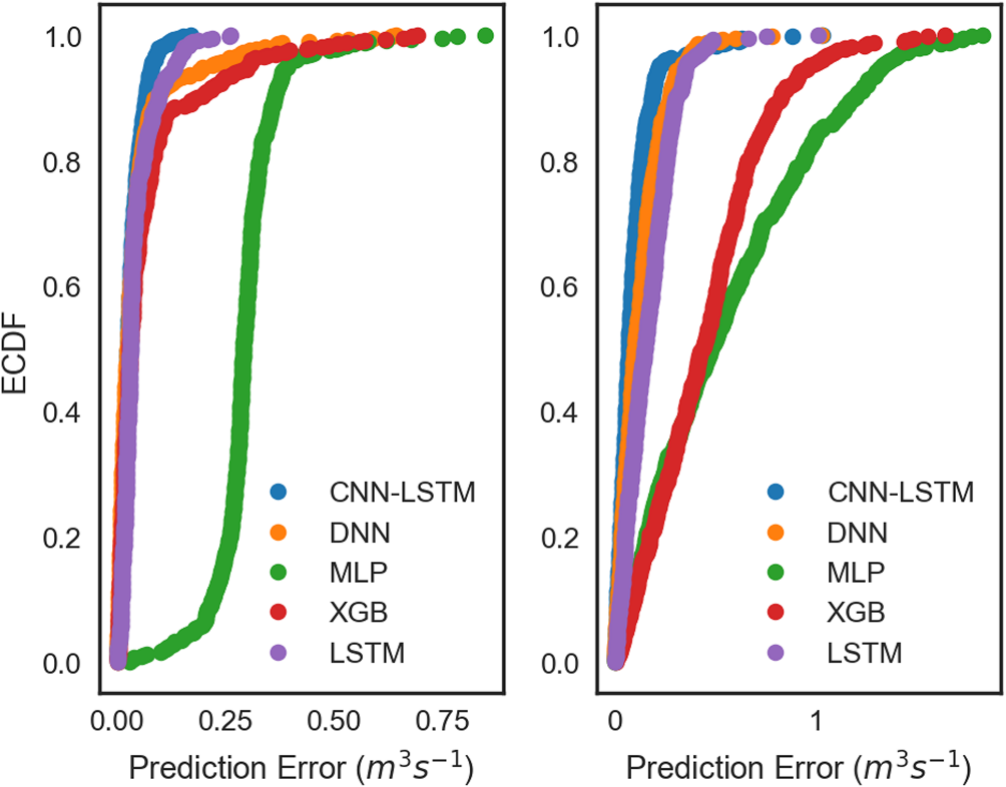
Empirical cumulative distribution function (ECDF) of absolute prediction error, |*PE*| (m^3^ s^−1^) of the testing data using CNN-LSTM vs. Deep Neural Network (DNN), Long Short Term Memory Network (LSTM), Multi-Layer Perceptron (MLP), and Extreme Gradient Boosting Regression (XGB) models in predicting streamflow (*Q,* m^3^ s^−1^) for Brisbane River (Left) and Teewah Creek (Right).

The empirical cumulative distribution function (ECDF, Fig. 12) at each site depicts the prediction capacity of different models. The proposed CNN-LSTM model is shown to be superior to the conventional AI and ensemble models as well as the standalone models including LSTM and DNN. Based on the error (0 to ± 0.05 m^3^ s^−1^) for both Brisbane River and Teewah Creek, Fig. 13 depicts that the proposed CNN-LSTM model is the most precise model in streamflow prediction.

**Figure 14:**
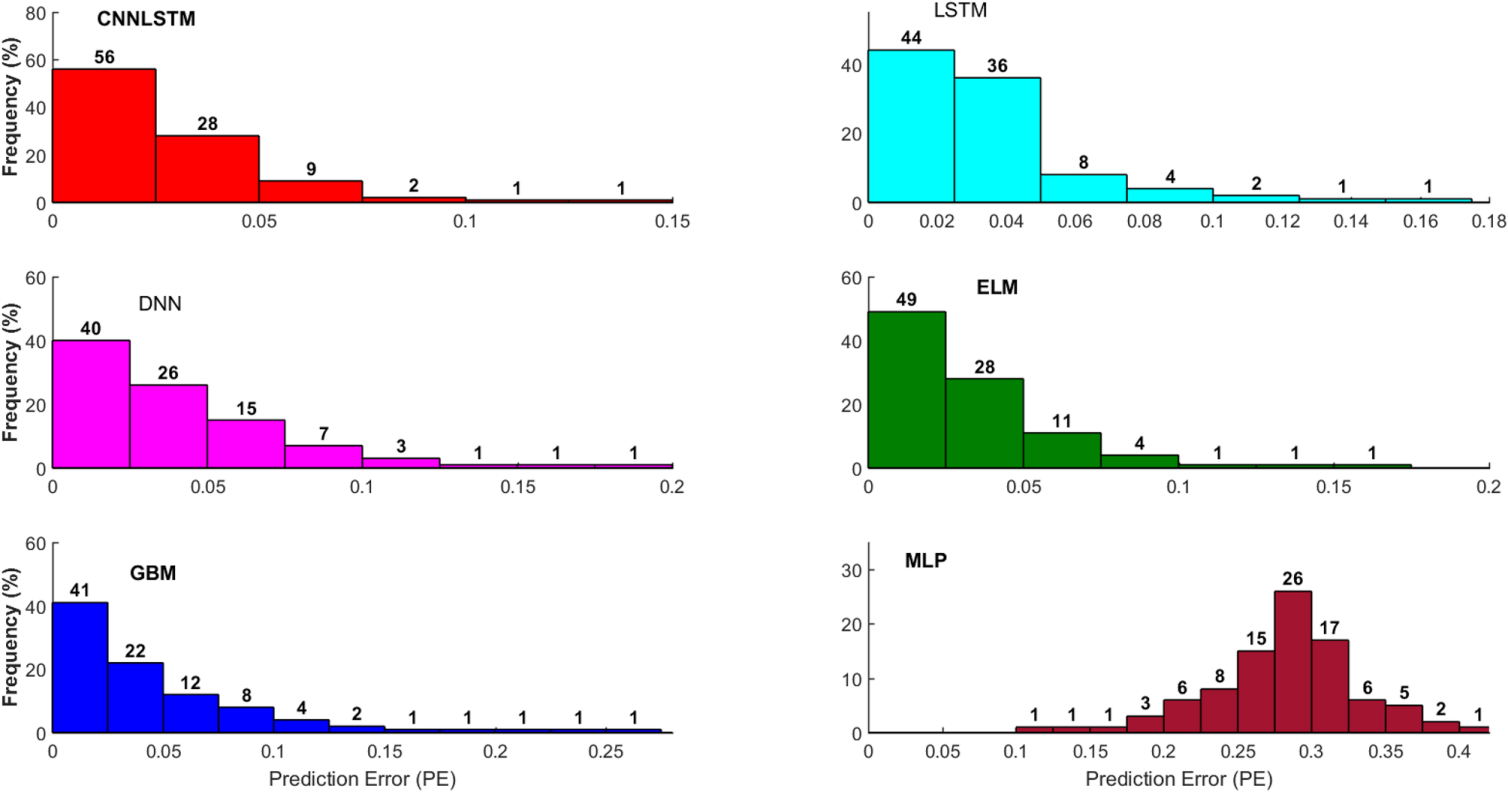
Histogram illustrating the frequency (in percentages) of absolute Prediction errors (|*PE*|, m^3^ s^−1^) of the best performing CNNLSTM model during test period (4-weeks) compared with Long Short-Term Memory Network (LSTM), Deep Neural Network (DNN), Extreme Learning Machine (ELM), Gradient Boosting Regression (GBM) and Multi-Layer Perceptron (MLP) model. for the prediction of hourly streamflow (*Q*, m^3^ s^−1^) at Brisbane River.

Figure 14 presents the frequency percentage distribution “histogram” of the predicted error (PE) based on the calculation of the error brackets with a 0.025 step size for Brisbane River. The presented graphical presentation can assist in a better understanding of model’s prediction performance^83^. The figure clearly reveals the outperformance of the CNN-LSTM model against the standalone models (DNN and LSTM), conventional AI models (MLP and ELM) and ensemble model (XGB), since its PE values are close to the zero frequency distribution. In a more quantitative term, the CNN-LSTM model shows the highest percentage of *PE* (56%) in the bin (0 < PE ≤ 0.025) followed by the ELM (49%), LSTM (44%), GBM (41%) DNN (40%), and finally the MLP model (0%). The accumulated PE percentages indicate that the PE of the CNN-LSTM model was below 0.15, while the conventional AI models yield a total of 97% and ensemble model yield a total of 89% of the PE in this band. This again supports the conclusion that CNN-LSTM is a superior technique for streamflow prediction.

To further investigate the prediction performance of the proposed CNN-LSTM model, the ${P}_{MAE} \; and \; {P}_{RMSE}$ of the experimental tests are employed to make the comparisons and analysis. Table 8 give the comparative analysis between the CNN-LSTM model and other involved models for the four experimental tests (1-Week, 2-Week, 4-Week and 9-Months). For instance, in 1-Week prediction, compared to LSTM model, the MAE and RMSE of CNN-LSTM model are reduced by 36.79% and 45.53% respectively for Brisbane River and 19.84% and 16.40% respectively for Teewah Creek. Similarly, reduction in MAE and RMSE of CNN-LSTM model compared to other model can be seen in 1-Week, 2-Weeks,4-Weeks, and 9-Months, hourly *Q*_*flow*_ prediction. There are no negative values in promoting percentage error, which indicates that the integration of CNN and LSTM model can derive better prediction accuracy.

**Table 8:** Promoting percentages of the comparison models [Convolutional Neural Network (CNN) and Long Short Term Memory Network (LSTM) as well as the Deep Neural Network (DNN), Multi-Layer Perceptron (MLP), Extreme Learning Machine (ELM), Gradient Boosting Regression (GBM), Decision Tree (DT), Multivariate Adaptive Regression Splines (MARS) and Extreme Gradient Boosting Regression (XGB) model] by the CNN-LSTM model for the testing period for 20% of total data (9-months), 1-Week (1W), 2-Week (2W) and 4-Week (4W) prediction.

	P_RMSE_ (%)								P_MAE_ (%)							
	Brisbane River				Teewah Creek				Brisbane River				Teewah Creek			
	20%	1W	2W	4W	20%	1W	2W	4W	20%	1W	2W	4W	20%	1W	2W	4W
CNN	40.26	34.82	43.69	41.75	38.31	44.11	43.69	29.98	75.07	42.38	41.61	54.00	44.59	45.61	41.61	35.73
DNN	32.22	13.88	21.93	12.05	48.00	18.28	21.93	21.60	62.21	14.28	7.68	27.04	44.53	14.93	7.68	17.84
DT	85.94	56.81	87.26	72.42	78.15	87.69	87.26	85.99	79.61	52.78	81.71	76.28	72.29	84.45	81.71	80.29
ELM	48.95	55.31	39.93	50.54	43.08	44.51	39.93	35.50	78.64	58.35	38.69	60.88	36.47	47.08	38.69	38.18
GBM	72.04	54.69	73.87	70.56	75.08	75.39	73.87	71.45	84.56	59.55	68.21	78.74	67.30	72.45	68.21	65.93
LSTM	16.03	36.79	23.55	23.60	13.08	19.84	23.55	25.02	71.00	45.53	13.70	41.86	14.71	16.40	13.70	17.61
MARS	62.13	54.84	87.26	73.61	44.76	87.69	87.26	85.99	52.89	41.56	81.71	76.97	31.82	84.45	81.71	80.29
MLP	56.62	63.87	88.20	75.93	60.73	59.98	88.20	64.56	92.55	69.66	87.20	81.58	84.56	58.64	87.20	75.31
XGB	72.79	54.13	73.99	70.47	74.57	75.81	73.99	71.62	84.23	59.10	68.91	78.60	67.14	73.55	68.91	67.28

P_MAE__=_ promoting percentages of mean absolute error and P_RMSE_ = promoting percentages of root mean square error.

**Figure 15:**
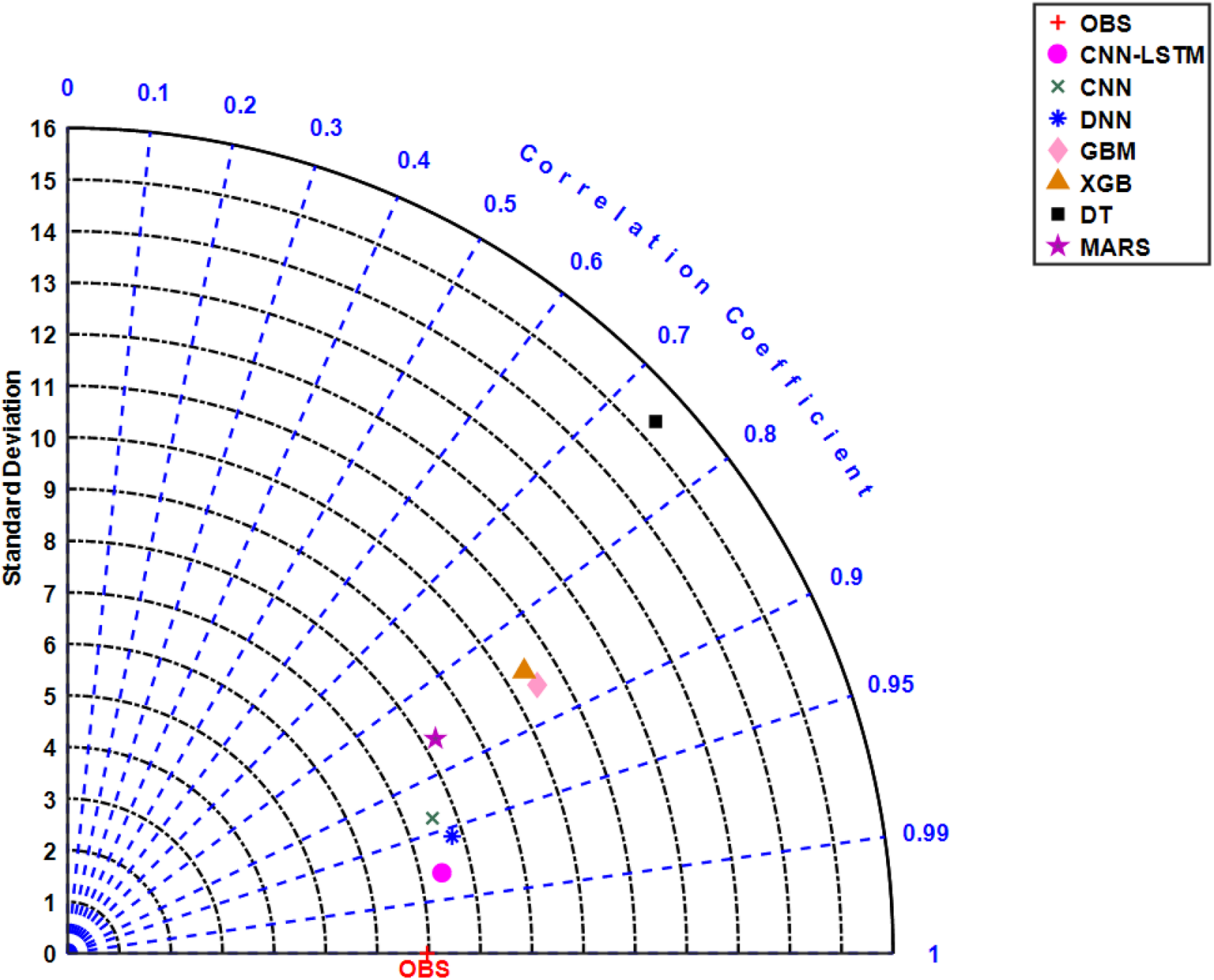
Taylor diagram showing the correlation coefficient between observed and predicted streamflow (*Q*, m^3^s^−1^) and standard deviation of CNN-LSTM, Convolutional Neural Network (CNN), Deep Neural Network (DNN), Multi-Layer Perceptron (MLP), Gradient boosting regression (GBM), Extreme Gradient Boosting Regression (XGB) model, Decision Tree (DT) and Multivariate Adaptive Regression Splines (MARS) during testing period (4-Week) for Brisbane River.

The model performance using Taylor diagram is presented in Fig. 15^105^. The main usage of this diagram is to present the closest predictive model with the observation in two-dimensional scale (standard deviation on the polar axis and correlation coefficient on the radial axis). Taylor diagram shows that the output of CNN-LSTM model is much closer to the actual observations compared to conventional AI and ensemble models.

Overall, the aforementioned evaluation results suggest that the CNN-LSTM model is superior to the standalone deep learning model as well as conventional AI and ensemble models. The proposed model CNN-LSTM is able to achieve a promising prediction performance and could be successfully applied to accurate and reliable hourly streamflow prediction. Furthermore, the averaged training time for the CNN-LSTM and the benchmarked models are listed in Table 2. Based on the results, DT followed by ELM, MARS and MLP requires the shortest training time but performs the worst in term of prediction accuracy. The proposed CNN-LSTM framework produces the most accurate prediction results with reasonable training time on various time horizons, including 1-Week, 2-Weeks, 4-Weeks and 9-Months.

## Conclusions and possible future research directions

This research investigated a new AI model based on the integration CNN with LSTM for modelling hourly streamflow at two different catchments of Australia (Brisbane River and Teewah Creek). The CNN network is employed to abstract the essential streamflow (*Q*_*flow*_) features while the LSTM is used for the prediction process based on the abstracted time series features. To validate the proposed CNN-LSTM prediction model, nine different well-established AI models (i.e., CNN, LSTM, DNN, MLP, ELM, GBM, XGB, DT, MARS) were also implemented. The construction of the proposed predictive model (i.e., CNN-LSTM) is designed based on six antecedent values recognised through statistical autocorrelation analysis of the streamflow data time series. Prediction has been established at different time intervals: 1-Week, 2-Weeks, 4-Weeks and 9-Months, which were evaluated based on graphical and statistical metrics. According to the attained prediction results, it can be concluded that:

- With low value of *RMSE* $\left(0.226\le RMSE\ge 0.155 \; \text{m}^{3}\;\text{s}^{-1} \left(Brisbane River\right)\right)$ and *MAE (*$0.196\le MAE\ge 0.054 \; \text {m}^{3}\; \text{s}^{-1} (Tewah Creek) )$ and high magnitude $[1.00\le WI\ge 0.996, 0.989\le LM\ge 0.868, 1.00\le {E}_{Ns\cdot }\ge 0.955 \left(Brisbane River\right) ]$ of the normalized index (*WI, LM* and *E*_*NS*_), CNN-LSTM model outperform the conventional AI as well as ensemble models;
- The streamflow prediction during testing phase in terms of *APB* and *KGE* were compared with the standalone deep learning models, conventional AI and ensemble models. The results revealed that CNN-LSTM (*KGE* ≥ *0.991 and APB* ≤ *0.527)* model is able to accomplish accurately prediction capacity in comparison with LSTM, CNN, DNN, MLP, ELM, XGB, GBM, DT and MARS models for both Brisbane River and Teewah Creek for all prediction intervals;
- With low normalized errors ($0.007\le NRMSE\le 0.028, 0.050\le RAE\le 0.132, 0.017\le NRMSE\le 0.020$), the CNN-LSTM model displays a better prediction accuracy against the comparative models in all the prediction intervals for both sites;
- With no negative value in promoting percentage error, the CNN-LSTM model demonstrates the best prediction accuracy ${P}_{MAE}=92.55\% \; and \; P_{RMSE}=56.62\%$ for the MLP model (Brisbane River, 9-Months *Q*_*flow*_ prediction);
- The hydrograph and scatter plot reveal that the prediction from the CNN-LSTM model is closer to the corresponding actual values with a minimum relative error ($RE\le 1.15 \; for \; CNN-LSTM, RE\le 2.69 \; for \; MLP)$ for peak flow values for both Brisbane River and Teewah Creek. In accordance to the error graphical presentation of boxplot, prediction error histogram and empirical cumulative distribution function confirmed the overall superior performance by the CNN-LSTM model with 84% of prediction error within 0–0.05 m^3^ s^−1^ range; Taylor plot of the compared models also reveals that the value of *r* for the CNN-LSTM model is closer to the actual *Q*_*flow*_ and this is evidencing the predictability performance capacity of the proposed model. All the above visualization results suggest that the CNN-LSTM model is the best model for *Q*_*flow*_ prediction in our comparison.

Future work could involve testing the CNN-LSTM model through integration of more casual hydrometeorological datasets (e.g., synoptic climate data or rainfall data) as an input predictor. During model development, the CNN-LSTM as well as other comparative models’ architecture that performed the best in the training period was determined as the optimal model (Table 2). However, the hyperparameter tuning methods like, Grid search^106^, Tree-structured Parzen estimators (Hyperopt)^107^, Population-based training^108^, Bayesian Optimization and HyperBand^109^ can also be used. These hyperparameter tuning methods can be time-consuming and resource-consuming, therefore separate study on the selection of best hyperparameter tuning methods can be conducted for *Q*_*flow*_ prediction. In addition, data uncertainty and non-stationarity can be investigated for further insights on their influence on the modeling predictability performance. Furthermore, research could also include the application of the CNN-LSTM model as new computer aid for watershed monitoring and management by incorporating a wider range of climate scenarios.
